# Wnt Effector TCF4 Is Dispensable for Wnt Signaling in Human Cancer Cells

**DOI:** 10.3390/genes9090439

**Published:** 2018-09-01

**Authors:** Dusan Hrckulak, Lucie Janeckova, Lucie Lanikova, Vitezslav Kriz, Monika Horazna, Olga Babosova, Martina Vojtechova, Katerina Galuskova, Eva Sloncova, Vladimir Korinek

**Affiliations:** 1 Institute of Molecular Genetics of the CAS, v. v. i., Videnska 1083, Prague 142 20, Czech Republic; lucie.janeckova@img.cas.cz (L.J.); lucie.lanikova@img.cas.cz (L.L.); vitezslav.kriz@img.cas.cz (V.Kr.); monika.horazna@img.cas.cz (M.H.); olga.babosova@img.cas.cz (O.B.); martina.vojtechova@img.cas.cz (M.V.); katerina.galuskova@img.cas.cz (K.G.); eva.sloncova@img.cas.cz (E.S.); 2 Faculty of Science, Charles University in Prague, Albertov 6, Praha 128 43, Czech Republic

**Keywords:** colorectal cancer, conditional gene inactivation, epithelium, gut, organoids, *TCF7L2*, tumorigenesis, Wnt signaling

## Abstract

T-cell factor 4 (TCF4), together with β-catenin coactivator, functions as the major transcriptional mediator of the canonical wingless/integrated (Wnt) signaling pathway in the intestinal epithelium. The pathway activity is essential for both intestinal homeostasis and tumorigenesis. To date, several mouse models and cellular systems have been used to analyze TCF4 function. However, some findings were conflicting, especially those that were related to the defects observed in the mouse gastrointestinal tract after *Tcf4* gene deletion, or to a potential tumor suppressive role of the gene in intestinal cancer cells or tumors. Here, we present the results obtained using a newly generated conditional *Tcf4* allele that allows inactivation of all potential Tcf4 isoforms in the mouse tissue or small intestinal and colon organoids. We also employed the clustered regularly interspaced short palindromic repeats (CRISPR)/Cas9 system to disrupt the *TCF4* gene in human cells. We showed that in adult mice, epithelial expression of Tcf4 is indispensable for cell proliferation and tumor initiation. However, in human cells, the TCF4 role is redundant with the related T-cell factor 1 (TCF1) and lymphoid enhancer-binding factor 1 (LEF1) transcription factors.

## 1. Introduction

The wingless/integrated (Wnt) signaling pathway represents one of the fundamental evolutionarily conserved signaling mechanisms controlling cell specification during embryonic development and in adult tissues. Aberrant activation of Wnt signaling causes a number of diseases, including various types of cancer [1]. Overall, there are at least five distinct branches of Wnt signaling. The best studied is the so-called canonical Wnt pathway, with β-catenin as its key effector [2]. Besides the structural function in adherens junctions, β-catenin accumulates in the cytoplasm of the Wnt ligand-stimulated cells, and it mediates Wnt signal transduction in the nucleus. Nuclear β-catenin associates with DNA-binding proteins of the lymphoid enhancer-binding factor/T-cell factor (LEF/TCF) family (further referred to as TCFs). β-Catenin converts TCFs from transcriptional repressors to activators, and TCF/β-catenin complexes upregulate the expression of Wnt target genes such as c*-myc*, *cyclin D1*, *CD44*, axis inhibition protein 2 (*Axin2*), and Sp5 transcription factor (*SP5*). In the absence of the Wnt stimulus, cytosolic β-catenin is marked for degradation by a cytoplasmic protein complex that includes casein kinase 1 alpha (CK1α) and glycogen synthase kinase 3 (GSK-3). The scaffolding of the kinases and β-catenin is mediated by axis inhibition protein 1/2 (Axin 1/2) and adenomatous polyposis coli (APC) tumor suppressors. Ultimately, N-terminally phosphorylated β-catenin is ubiquitinated and subsequently destroyed by the proteasome. Wnt signaling is activated by extracellular Wnt ligand binding to a receptor complex that is composed of a seven-transmembrane receptor of the Frizzled (Fz) family, and co-receptor low density lipoprotein receptor-related protein 5/6 (LRP5/6). The ligand-receptor engagement triggers a cascade of events that induce the phosphorylation of adaptor protein Dishevelled (Dvl), and eventually results in inhibition (or partial disassembly) of the β-catenin destruction complex, and β-catenin accumulation. Besides TCFs, β-catenin interacts with several other transcription factors including androgen receptor (AR), liver receptor homolog 1 (LRH1), hypoxia-induced factor 1 alpha (HIF1α), and SRY-box 17 (Sox17) [2].

Many studies have documented that the canonical Wnt pathway regulates the proliferation and potency of intestinal stem cells (ISCs). For instance, the genetic disruption of the pathway’s effectors β-catenin (encoded by the *Ctnnb1* gene) or Tcf4 (encoded by the *Tcf7l2* gene; for the sake of clarity, the term Tcf4 will be used for both the Tcf4 protein and gene throughout the study), is associated with the demise of small intestinal crypts. Conversely, aberrant activation of the Wnt pathway increases the stem cell numbers, and initiates intestinal tumorigenesis [3,4]. Interestingly, some ISC-specific markers such as leucine-rich repeat-containing G-protein-coupled receptor 5 (LGR5) [5,6], or tumor necrosis factor receptor superfamily, member 19 (TNFRSF19 or TROY) [7] are encoded by the Wnt signal-responsive genes.

The epithelial lining of the gastrointestinal tract renews every 3–5 days, representing one of the most intensively self-replenishing organs in mammals [8]. The monolayer of both the small intestinal and colonic epithelium penetrates into the underlying connective tissue of lamina propria to form tubular glands called the crypts. The crypt bottom is populated by multipotent ISCs that maintain tissue homeostasis. The cells divide approximately every 24 h, generating a pool of transit amplifying (TA) progenitor cells, rapidly proliferating cells that migrate upwards the crypt axis. At the crypt orifice, TA cells differentiate to several cell types that mainly include absorptive enterocytes, mucus-producing goblet cells, or hormone-releasing enteroendocrine cells. In the small intestine, the differentiated cells cover the villi, which are luminal protrusions of the mucosa that increase the epithelial surface. The surface area of the large intestine occupied by differentiated cells, which also covers the upper third of the crypts, is flat. The differentiated cells are shed from the epithelial layer; this mechanism ensures constant cell renewal of the tissue in the harsh environment of the gastrointestinal (GI) tract lumen. The small intestinal epithelium is also protected by bactericidal Paneth cells that do not migrate from the crypt, but stay at the crypt bottom, where they persist for six to eight weeks [9].

Colorectal carcinoma (CRC), i.e., cancer affecting the colon and rectum, represents one of the most often diagnosed neoplasia in developed countries [10]. It is presumed that in colorectal tumors, the first oncogenic mutation provides selective advantage to the epithelial cell, which multiplies and forms a (micro)adenoma. In the majority (>80%) of sporadic colorectal tumors, the “initiatory” mutations frequently occur in the *APC* gene encoding the negative regulator of canonical Wnt signaling. Consequently, the APC-inactivating mutations aberrantly activate the Wnt pathway, even in the absence of the external Wnt signal [11]. It has been documented that in some CRCs, hyperactive Wnt signaling might result from mutations affecting additional pathway negative regulators AXIN1 [12] and AXIN2 [13], or upon missense mutations in the *CTNNB1* gene that impair β-catenin protein N-terminal phosphorylation [14]. In all the above examples, pathological transformation of the gut epithelium is driven by stabilized β-catenin that mediates inappropriate transcriptional activation of TCF/β-catenin-responsive genes [15]. Intriguingly, the results of whole exome/genome sequencing of genomic DNA isolated from CRC specimens brought a somewhat different view of the role of the Wnt pathway (or its individual components) in CRC pathogenesis. Analysis of more than 200 CRC specimens revealed that the *TCF4* gene was inactivated in 31% of microsatellite-unstable (MSI) and 12% of microsatellite-stable (MSS) cancers. Moreover, the *TCF4* locus was deleted in a subset of the examined cases [16]. These loss-of-function mutations imply that apart from its physiological role in healthy intestines (see further), the *TCF4* status is important for the initiation and/or progression of CRC. Additionally, a genome-wide RNA-mediated interference (RNAi) screen identified TCF4 as a transcriptional repressor, decreasing the Wnt pathway output and restricting CRC cell growth [17].

It is presumed that in the mouse intestine, Tcf4 is crucial for embryonic development and adult tissue homeostasis of the small intestinal and colonic epithelia [18]. However, some results of the Tcf4 targeting experiments are contradictory. The Tcf4 whole-body knockout generated by the insertion of the expression cassette producing hygromycin B phosphotransferase immediately upstream of the high mobility group (HMG) DNA binding domain sequence (so-called HMG box; we designated the modified allele *Tcf4^Hyg^*) was perinatal lethal due to the absence of the proliferative compartments in the small intestine. However, the colon epithelium in these Tcf4-deficient mice stayed seemingly intact [19]. Conditional Tcf4 inactivation performed by Cre-mediated excision of the floxed sequence encoding the HMG box (we named the modified allele *Tcf4^floxHMG^*) impaired cell proliferation in both the adult small intestine and the colon [20]. In contrast, *Tcf4* gene-driven Cre recombinase-mediated deletion of the first *Tcf4* exon (modified allele: *Tcf4^flox1^*) induced hyperproliferation of progenitor cells in the small intestinal and colonic epithelium at embryonic day (E) 13.5. This resulted in cell exhaustion, accompanied by the disruption of the small intestinal and colonic architecture at E14.5. Moreover, *Tcf4* haploinsufficiency promoted the formation of colonic tumors in multiple intestinal neoplasia (Min) mice, which represent the mouse model of intestinal tumorigenesis initiated by Apc loss [21]. In addition, using the same, i.e., *Tcf4^flox1^*, allele Angus-Hill and colleagues observed that complete knockout of Tcf4 in the adult colon resulted in the formation of aberrant crypt foci (ACF), which are considered to be the earliest neoplastic lesions during CRC initiation [22]. The latter findings indicated—similarly to the situation observed in human CRC—the tumor-suppressive function of Tcf4.

To address these contradictory results, we employed a newly generated mouse strain harboring exon 5 flanked by *loxP* sites (allele name: *Tcf4^flox5^*). The exon is included in all annotated transcripts in the human and mouse *Tcf4* gene, and in addition, it is positioned downstream from the most frequently used transcription start sites that initiate mRNAs encoding either a full-length or truncated version of the protein lacking the β-catenin binding domain [18]. As Cre-mediated excision of exon 5 leads to an open reading frameshift, production of the majority of the Tcf4 protein variants is abolished. The *Tcf4^flox5^* allele was used to inactivate Tcf4 in embryonic and adult mouse intestinal epithelia using several different Cre drivers. Tcf4 deletion was also performed in organoids derived from the small intestine and colon. In addition, we employed the clustered regularly interspaced short palindromic repeats (CRISPR)/Cas9 system to disrupt the *TCF4* gene and its closest paralog *TCF3* (alias *TCF7L1*) in human cells, and tested the effect(s) of the disruption on cell viability and Wnt pathway-driven transcription. Our results indicate the importance of mouse Tcf4, mainly in adult intestinal epithelium homeostasis and intestinal tumor initiation. In contrast, in human cells, the TCF4 function is substituted by other LEF/TCF family members.

## 2. Materials and Methods

### 2.1. Experimental Mice

Housing of mice and in vivo experiments were performed in compliance with the European Communities Council Directive of 24 November 1986 (86/609/EEC), and national and institutional guidelines. The Animal Care Committee of the Institute of Molecular Genetics approved the animal care and experimental procedures (Ref. Nos.: 63/2013, 30/2017, and 58/2017). *Tcf4^lacZ/+^* mice were purchased from the European Conditional Mouse Mutagenesis Program (EUCOMM; Wellcome Trust Sanger Institute; strain: *Tcf7l2^tm1a(EUCOMM)Wtsi^*). To generate *Tcf4^flox5/flox5^* mice, the *lacZ-neomycin phosphotransferase* expression cassette flanked by FTR sites was removed from the genome by mating *Tcf4^lacZ/+^* with ACTB-FLPe (purchased from the Jackson Laboratory, Bar Harbor, ME, USA) transgenic mice. Mice of the ACTB-FLPe strain express enhanced FLP recombinase (FLPe) from the ubiquitously active regulatory region of the human *β-ACTIN* gene [23]. *Apc^flox14/flox14^* mice were purchased from the Mouse Repository (National Cancer Institute, Frederick, MD, USA); *Villin-Cre* and *Villin-CreERT2* animals [24] were kindly provided by S. Robine (Institut Curie, Centre de Recherche, Paris, France). *Lgr5-EGFP-IRES-CreERT2* [B6.129P2-Lgr5^tm1(cre/ERT2)Cle/J^], *Rosa26R-lacZ* [B6;129S4-Gt(ROSA)26^Sortm1Sor/J^], and *Rosa26R-tdTomato* [B6;129S6-Gt(ROSA)26^Sortm14(CAG−tdTomato)Hze/J^] mice were purchased from the Jackson Laboratory. Animals were housed in specific pathogen-free (SPF) conditions, and genotyped according to the provider’s or published protocols. For Cre-mediated recombination, mice were administered using an intraoral gavage of tamoxifen (Sigma-Aldrich, St. Louis, MO, USA; stock 10 mg/mL in ethanol). Prior to gavage, the tamoxifen solution was mixed with mineral oil (Sigma-Aldrich). For tumor initiation experiments, mice were administered with a single dose containing 1 mg of tamoxifen; 5 mg of tamoxifen per dose was used in all other experiments.

### 2.2. Cell and Organoid Culture; 4-Hydroxytamoxifen Treatment

SW480 were obtained from the American Type Culture Collection; M1 cells were generated previously [25]. SuperTOPFlash HEK293 (STF) cells were a kind gift of Q. Xu and J. Nanthas (Jonhns Hopkins University, Baltimore, MD, USA; [26]). Cells were maintained in Dulbecco’s Modified Eagle’s Medium (DMEM; Sigma-Aldrich) supplemented with 10% fetal bovine serum (FBS; Thermo Fisher Scientific, Waltham, MA, USA) and streptomycin, penicillin, and gentamicin mix (Thermo Fisher Scientific). Cells in cultures were checked for the presence of mycoplasma infection using the MycoAlert kit (Lonza, Basel, Switzerland). TCF3- and TCF4-deficient SW480 cells were generated by the CRISPR/Cas9 system; single guide RNA (sgRNA) targeting the first exon of *TCF3* or *TCF4* was designed using the CRISPR DESIGN tool (crispr.mit.edu). Corresponding oligonucleotides (Table S1) were cloned into the LentiGuide-Puro vector (Addgene #52962); cells were co-transfected with the LentiCas9-Blast (Addgene #52963; [27]) and pARv-RFP vectors (Addgene #60021). The latter vector was used for single-cell sorting of prospective recombined clones, as described in [28]. Successfully edited clones (containing frameshift mutations in both alleles) were selected by PCR/restriction endonuclease AvaI (TCF3)/PvuII (TCF4; both enzymes were purchased from Thermo Fisher Scientific) and sequencing of the locus. Cell viability was determined using the AlamarBlue Cell Viability Assay (Thermo Fisher Scientific). The reaction was measured in five replicates (for each cell line and time point) using an EnVision 2105 Multimode Plate Reader (Perkin Elmer, Waltham, MA, USA). Organoid cultures were established from freshly isolated intestinal crypts as described previously [29,30]. Briefly, the middle part of the small intestine was longitudinally cut, villi were scraped off by a cover glass, and the tissue was repeatedly washed in phosphate-buffered saline (PBS); the colon was cut longitudinally and washed several times in PBS. Tissues were incubated with 5 mM pH 8 ethylenediaminetetraacetic acid (EDTA) solution (Sigma-Aldrich) at 4 °C for 30 min (small intestine) or 90 min (colon), and then shaken gently to remove the remaining differentiated epithelial cells. The tissue was transferred to fresh PBS and shaken well to release the crypts to the supernatant. The supernatant was filtered through a 70-μm strainer (Corning; Corning, NY, USA). Pelleted crypts (180 × *g* at 4° for 5 min) were washed in advanced DMEM/F-12 medium (Thermo Fisher Scientific), transferred to Matrigel (BD Biosciences, San Jose, CA, USA), and maintained in advanced DMEM/F-12 medium supplemented with 1 × N-2 supplement (Thermo Fisher Scientific), 1 × B-27 supplement (Thermo Fisher Scientific), mRspo1 (500 ng/mL; Peprotech, Rocky Hill, NJ, USA), mNoggin (100 ng/mL; Peprotech), 10mM 4-(2-hydroxyethyl)-1-piperazineethanesulfonic acid (HEPES, Thermo Fisher Scientific), 1 × Glutamax (Thermo Fisher Scientific), 1 mM N-acetyl-cysteine (Sigma-Aldrich), 1 × Penicillin/Streptomycin (Thermo Fisher Scientific), mouse Epidermal growth Factor (mEGF, 50 ng/mL; Thermo Fisher Scientific), primocin (100 μg/mL, Invivogen, Toulouse, France). Medium for colon organoids was further supplemented with Wnt3a-conditioned medium (dilution 1:1; Wnt3a-producing cells were kindly donated by M. Maurice, University Medical Center Utrecht, Utrecht, The Netherlands). Cre-mediated recombination in the organoids was induced by adding 4-hydroxytamoxifen (4-OHT) (Sigma-Aldrich; final concentration 2 μM, 1 mM stock solution was prepared in ethanol) to the culture media.

### 2.3. Cell Transfection, Small Interfering RNA, and Immunoblotting

Cells were transfected using Lipofectamine RNAiMAX or Lipofectamine 2000 (Thermo Fisher Scientific) according to the manufacturer’s protocol; small interfering RNA (siRNA) targeting *TCF1* (alias *TCF7*; M-019735-00-0020), *LEF1* (M-015396-00-0020), and control non-silencing siRNA (D-001206-13-20) were purchased from Dharmacon; siRNA against β-CATENIN (*CTNNB1*; s438) was obtained from ThermoFisher Scientific. Cells were transfected twice in a 2-day interval, and harvested 24 h after the second transfection. The region encoding the N-terminal portion of *Tcf4* gene (N-Tcf4) was amplified from mouse complementary cDNA and cloned into the pK-Myc expression vector [31]; cloning primers are listed in Table S1. Luciferase assays were performed as described previously [32] using the Luciferase/Renilla assay system and GloMax 20/20 luminometer (all from Promega). Control pRL-TK reporter was purchased from Promega. The SuperTOPFlash and SuperFOPFlash reporters [33] were kindly provided by R. Moon (University of Washington School of Medicine, Seattle, WA, USA). The assay was performed in SW480 cells. The transfection mix contained SuperTOPFlash reporter (control transfections included the SuperFOPFlash reporter with mutated TCF binding sites), increasing amounts of N-Tcf4-pK-Myc, and the transfection efficiency control pRL-TK vector. All luciferase assays were performed in triplicates, and the resulting average values together with standard deviations (SDs) were calculated from three independent experiments after normalization to Renilla luciferase values. Immunoblotting was performed according to the protocol published previously [32] using the following primary antibodies: anti-TCF1 (#2203, Cell Signalling; Danvers, MA, USA), anti-LEF1 (#2230, Cell Signalling), anti-TCF3 (#2883, Cell Signalling), anti-TCF4 (#2569, Cell Signalling), and anti-CTBP (sc-17759, Santa Cruz Biotechnology, Santa Cruz, CA, USA). Peroxidase-conjugated anti-rabbit (#7074, Cell Signalling) and anti-mouse (#7076, Cell Signalling) secondary antibodies were used to obtain the bioluminescent signal.

### 2.4. RNA Isolation and Quantitative Reverse Transcription-Polymerase Chain Reaction (qRT-PCR)

Total RNA from cells grown in cultures was isolated using TRI Reagent (Sigma-Aldrich) and reverse-transcribed using RevertAid Reverse Transcriptase (Thermo Fisher Scientific) according to the manufacturer’s protocol. For sorted cells and organoids, RNeasy Micro Kit (Qiagen, Hilden, Germany) and MAXIMA Reverse Transcriptase (Thermo Fisher Scientific) were used. qRT-PCR was performed in triplicates using the SYBR Green I Master Mix and LightCycler 480 apparatus (Roche, Basel, Switzerland). Primers are listed in Table S1.

### 2.5. Fluorescent Microscopy

Fluorescent staining of small intestinal organoids was performed as follows. Matrigel-embedded organoids were washed with PBS and fixed in 4% (*w*/*v*) paraformaldehyde (Electron Microscopy Sciences, Hatfield, PA, USA) in PBS for 30 min at room temperature (RT). After fixation, organoids were washed in PBS and incubated with 0.1% Triton X100 (Sigma-Aldrich) in PBS for 30 min. After additional washing in PBS, organoids were incubated in 5% goat serum (Vector Laboratories, Burlingame, CA, USA) in PBS and washed again. Incubation with primary anti-Tcf4 antibody (#2569, Cell Signalling) was performed overnight at 4 °C. After washing (PBS), the organoids were incubated (2 h at RT) with a goat anti-rabbit immunoglobulin Alexa Fluor 488-conjugated secondary antibody (Thermo Fisher Scientific); cell nuclei were counterstained with diamidino-2-phenylindole (DAPI; 0.1 μg/mL, Sigma-Aldrich) for 10 min at RT. Stained organoids were kept (and photographed) in Scaleview-A2 optical clearing agent (Olympus) at 4 °C for 1 week. Fluorescent pictures were acquired using spinning disk confocal microscope Dragonfly (Andor) and processed using Imaris software (Bitplane, Belfast, UK).

### 2.6. Fluorescence-Activated Cell Sorting

The middle portion of the small intestine or the whole colon was cut longitudinally, washed in PBS, and incubated in 5 mM pH 8 EDTA solution in PBS (Sigma-Aldrich) at 4 °C for 30 or 60 min, respectively. Epithelial cells were obtained by incubation with dispase (Thermo Fisher Scientific; stock solution 100 mg/mL diluted 1:300 in serum-free DMEM) using rigorous shaking of the tissue pieces on rotating platform (800 RPM, 3 × 5 min, 37 °C). Supernatant was diluted in DMEM containing 10% FBS, and passed through a 40-μm strainer (Corning) to obtain a single-cell suspension. Cells were stained with allophycocyanin (APC)-conjugated anti-epithelial cell adhesion molecule (EpCam; dilution 1:500; 17-5791-80, Thermo Fisher Scientific) and phycoerythrin (PE)-conjugated anti-CD24 (1:200; 12-0242-81, Thermo Fisher Scientific) primary antibodies for 30 min at 4 °C. Cells were gated by forward scatter (FSC), side scatter (SSC), and negative staining for Hoechst 33258 (Sigma-Aldrich). EpCam^+^ epithelial cells from the small intestine of the *Lgr5-EGFP-IRES-CreERT2* mouse were sorted according to CD24 and green fluorescent protein (GFP) expression to CD24^−^/GFP^−^ (differentiated cells), CD24^+^/GFP^−^ (Paneth cells), and CD24^+^/GFP^+^ (crypt base cells). Based on the SSC signal, the CD24^+^/GFP^+^ population was further divided into CD24^+^/GFP^+^ large cells (precursors of the intestinal secretory cell lineage) and CD24^+^/GFP^+^ small cells (ISCs). Cells sorting was performed using an Influx cell sorter (BD Biosciences).

### 2.7. Immunohistochemistry and β-Galactosidase Staining

Intestines were fixed in 4% formaldehyde (*v*/*v*; Sigma-Aldrich) in PBS overnight, and embedded in paraffin using an automatic tissue processor (Leica, Wetzlar, Germany); 5-µm sections were stained according to the protocol published previously [34]. Briefly, specimens were deparaffinized in xylene, and antigen retrieval was performed in a steam bath by immersing the slides into 10 mM citrate buffer pH 6.0. Endogenous peroxidase activity was blocked by incubation in 0.2% H_2_O_2_ (Sigma-Aldrich; stock 30%) in methanol (Merck, Kenilworth, NJ, USA) for 20 min. Specimens were incubated overnight at 4 °C with primary antibodies: anti-PCNA (ab18197, Abcam, Cambridge, UK), anti-TCF3 (sc-8635, Santa Cruz Biotechnology), and anti-TCF4 (#2569, Cell Signalling). Subsequently, biotin-conjugated secondary antibodies were used (Biotin-XX Goat anti-Rabbit IgG, B-2770, Thermo Fisher Scientific; Biotin-XX Rabbit anti-Goat IgG, #31732, Thermo Fisher Scientific). The signal was enhanced using Vectastain ABC kit (Vector Laboratories). The slides were developed in 3,3′-diaminobenzidine (DAB; Sigma-Aldrich; 30 mg dissolved in 90 mL 50 mM Tris, pH 7.5) supplemented with 0.3% H_2_O_2_ (*v*/*v*; Sigma-Aldrich). Sections were counterstained with hematoxylin (PENTA). β-galactosidase was visualized using the 5-bromo-4-chloro-3-indolyl-β-d-galactopyranoside (X-gal; Sigma-Aldrich) substrate, followed by counterstaining using nuclear fast red stain (DiaPath) [34]. Periodic acid Shiff (PAS) staining was performed using the P.A.S. acc. Hotchkiss-Mc Manus kit (DiaPath) according to the manufacturer’s instructions.

### 2.8. Statistical Analysis

The results of the quantitative reverse transcription polymerase chain reaction (qRT-PCR) and β-Galactosidase (lacZ) staining were evaluated by a Student’s *t*-test.

## 3. Results and Discussion

### 3.1. Analysis of Tcf4 Expression in the Mouse Small Intestine and Colon

**Figure 1:**
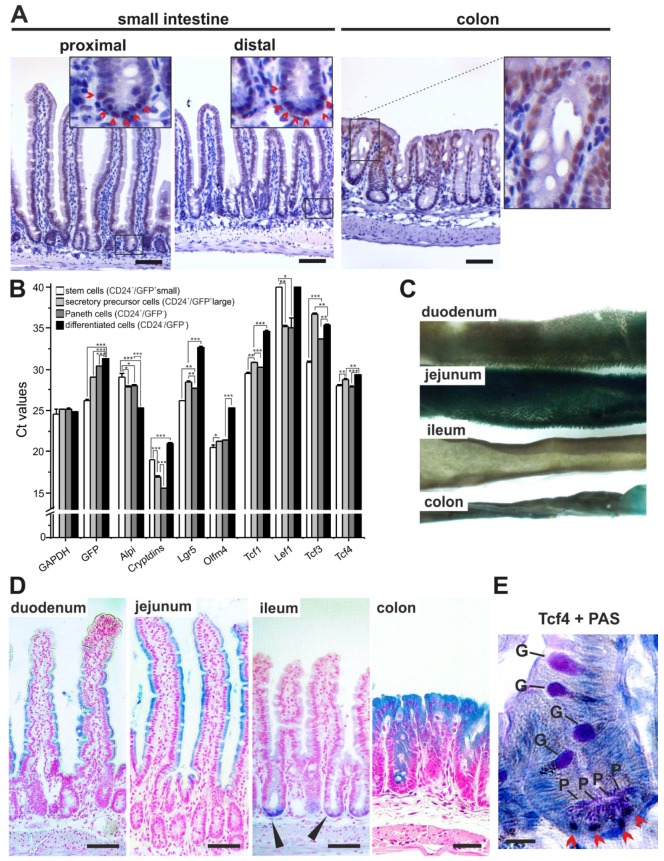
Differential pattern of *Tcf4* expression in the mouse small intestine and colon. (**A**) Immunohistochemical detection of endogenous Tcf4 in the proximal and distal parts of the small intestine and colon. The Tcf4 nuclear positivity is indicated by 3,3′-diaminobenzidine (DAB) stain (brownish nuclear precipitate). The specimens were counterstained with hematoxylin (blue nuclei). Magnified images are shown in the insets. Tcf4-positive cells were detected in the crypts of both proximal and distal parts of the small intestine (red arrowheads), and in the upper portion of the colonic epithelium. (**B**) Quantitative reverse transcription polymerase chain reaction (qRT-PCR) analysis of *Lef/Tcf* gene expression in cell populations isolated from the small intestinal crypts of *Lgr5- CreERT2* mice. Cells were sorted according to their size and green fluorescent protein (GFP) or cluster of differentiation 24 (CD24) positivity to stem cells (CD24^+^/GFP^+^ small cells), secretory precursor cells (CD24^+^/GFP^+^ large cells), Paneth cells (CD24^+^/GFP^−^ cells) and differentiated cells (CD24^−^/GFP^−^ cells). The diagram shows the threshold cycle (Ct) values normalized to *β-actin* (*Actb*) gene expression (the *Actb* gene Ct value was arbitrarily set to 21); *GAPDH* was—next to *Actb*—an additional housekeeping gene. The analysis was performed on cell populations obtained from three animals of the corresponding genetic background; qRT-PCR reactions were run in technical triplicates. Results of one representative experiment are shown; error bars indicate standard deviations (SDs); * *p* < 0.05; ** *p* < 0.01; *** *p* < 0.001 (Student’s *t*-test). *Cryptdins* indicate the expression of cryptdin 1,3,6–12,14,15 detected by a common primer pair. (**C**) Stereomicroscopy images of whole-mount detection of β-galactosidase (lacZ) in the intestine of *Tcf4^lacZ/+^* reporter mice. Specimens were developed using the 5-bromo-4-chloro-3-indolyl-beta-d-galactopyranoside (X-gal) substrate (blue stain). (**D**) Sections showing the lacZ activity on the villi of the duodenum and jejunum, in the crypts of the ileum (black arrowheads), and in the cells lining the colonic surface. Specimens were counterstained with fast nuclear red. (**E**) Immunodetection of nuclear Tcf4 (DAB stain; red arrowheads) in periodic acid-Schiff (PAS)-stained Paneth cells (P). Notice the PAS positivity (dark violet stain) in maturating goblet cells (G). The section was generated from the ileum of *Tcf4^lacZ/+^* mice upon lacZ visualization. Scale bar: (**A**,**C**) 0.1 mm; (**E**) 5 μm.

To survey Tcf4 expression in the adult small intestine and colon, we used immunohistochemistry (IHC) detection of the Tcf4 protein. Staining of paraffin-embedded sections with a Tcf4-specific antibody revealed an expression pattern that was similar to results published previously [35]. In the small intestine, the Tcf4 positive result was noticed throughout the epithelium, with slightly less pronounced staining in the upper part of the crypts, where rapidly dividing TA cells are localized. The staining pattern was reproduced irrespective of the position along the rostro-caudal axis of the organ. In contrast, in the colon, the strongest Tcf4 nuclear positivity was noted in differentiated cells located on the colon surface (Figure 1A). To assign Tcf4 expression to individual cell types present in the small intestinal crypts, we isolated epithelial crypt cells from *Lgr5-EGFP-IRES-CreERT2* (further referred to as *Lgr5-CreERT2*) mice producing enhanced green fluorescent protein (EGFP) and CreERT2 fusion proteins in ISCs and secretory cell precursors [6,36]. The green fluorescent signal and anti- cluster of differentiation 24 (CD24) surface labeling (the labeling marks epithelial cells located at the lower portion of the crypts [37]) was used to discriminate the differentiation status of epithelial cells. Total RNA obtained from the sorted cell populations was employed for qRT-PCR analysis of all TCFs. The expression levels of intestinal alkaline phosphatase (*Alpi*) and cryptdins were used as markers of differentiated enterocytes or Paneth cells, respectively, and *Olfm4* was used as an additional ISC marker. We observed a slight discrepancy in the level of EGFP and Lgr5 expression in Paneth cells and secretory precursors, probably caused by the mosaic production of EGFP from the knock-in allele [38]. Nevertheless, our analysis showed that the highest levels of *Tcf4* mRNA were detected in the cells present at the crypt bottom, especially in the ISCs and Paneth cells. We anticipate that the qRT-PCR analysis precluded direct comparison of the mRNA levels among different genes; nevertheless, in agreement with published data, (relatively) high expression of *Tcf1* in ISCs was observed (Figure 1B). We also employed the *Tcf4^lacZ/+^* reporter strain generated by the knock-in of the lacZ expression cassette into the *Tcf4* locus, to follow Tcf4 intestinal expression. As expected, the lacZ-positivity phenocopied Tcf4 immunohistochemical detection. The only noticeable difference was the absence of blue staining in the crypts of the duodenum and jejunum, and in the villus cells of the ileum. Consequently, lacZ production was undetectable in whole-mount specimens of the ileum (Figure 1C, D). Since the lacZ enzyme is produced from one allele only, we suggest that the discrepancy between the two staining methods was caused by lower gene dosage, resulting in the decreased sensitivity of the lacZ enzyme detection. Interestingly, PAS-mediated visualization of Paneth and Goblet cells, combined with anti-Tcf4 staining, revealed that in the ileal crypts, the highest, i.e., detectable, levels of Tcf4 proteins were produced in Paneth cells (Figure 1E; figure legend is on the next page).

### 3.2. Intestinal Epithelium-Specific Tcf4 Inactivation Had No Effect on the Embryonic Gut

**Figure 2:**
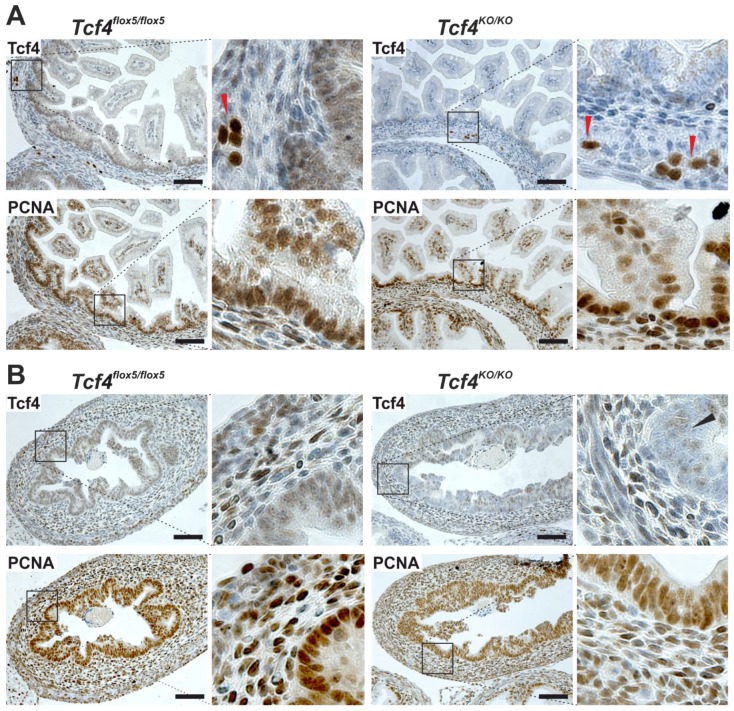
Normal development of the embryonic gut upon Tcf4 inactivation. Immunohistochemical analysis of the small intestine (**A**) and colon (**B**) of *Tcf4^flox5/flox5^* and *Tcf4^flox5/flox5^ Villin-Cre* (*Tcf4^KO/KO^*) mice at embryonic day (E) 17.5. Notice the Tcf4 nuclear positivity in the intervillus regions of *Tcf4^flox5/flox5^* mice that is missing in *Tcf4^KO/KO^* animals. The regions retain their proliferation, as evidenced by proliferating cell nuclear antigen (PCNA) staining. The colon is less developed; however, no obvious phenotype related to the Tcf4 loss is visible. Inactivation of the floxed *Tcf4* alleles in the colon is incomplete, since groups of Tcf4-positive cells are present in the tissue (black arrowhead). In mice of both genotypes, Tcf4-positive cell nuclei are also detected in the small intestinal enteric plexus (red arrow heads) and in the subepithelial layers of the colon. Specimens were counterstained with hematoxylin. Magnified images are shown at the right. Scale bar: 0.3 mm.

Next, we intercrossed *Tcf4^flox5/flox5^* mice with *Villin-Cre* transgenic mice; the latter mice produce constitutively active Cre driven by the murine *villin* promoter. The transgene is active in epithelial cells of the small intestine and colon [24]. Interestingly, no obvious phenotype was observed in the developing gut. Despite Tcf4 absence, the intervillus regions of the small intestinal epithelium at embryonic day (E) 17.5 contained proliferating cell nuclear antigen (PCNA)-positive cells. (Figure 2A). The observed phenotype was in sharp contrast to previously documented phenotypes of the mouse models of *Tcf4* gene inactivation using *Tcf4^Hyg/Hyg^* animals [19], or by conditional deletion of the *Tcf4^floxHMG^* alleles using *PGK-Cre* transgenic animals (genotype: *PGK-Cre Tcf4^floxHMG/floxHMG^*) [20]. The obvious explanation for the observed discrepancy would be incomplete inactivation of “our” *Tcf4^flox5^* allele. It was documented that the *Villin-Cre* transgene is active in the entire intestinal epithelium at E12.5, i.e., several days prior to transition from pseudostratified to columnar epithelium [24]. Contrary to the original description, we observed groups of Tcf4-positive cells in the colon epithelium of *Tcf4^flox5/flox5^ Villin-Cre* embryos at E17.5, indicating less efficient Cre-mediated DNA recombination (Figure 2B). Nevertheless, the small intestinal epithelium appeared to be recombined “completely”, excluding the possibility that the absence of the small intestinal phenotype was caused by partial recombination. The *PGK* promoter drives ubiquitous expression of the Cre enzyme [39]. Consequently, the situation in *PGK-Cre Tcf4^floxHMG/floxHMG^* animals mimics Tcf4 whole-body inactivation achieved in *Tcf4^Hyg/Hyg^* mice. We hypothesized that the Tcf4 function in the cells outside of the epithelium might contribute to the small intestinal defect manifested in *Tcf4^Hyg/Hyg^* and *PGK-Cre Tcf4^floxHMG/floxHMG^* animals. Interestingly, we observed Tcf4 nuclear positivity in subepithelial layers of developing colon tissue at E17.5 (Figure 2B). In the small intestine, the non-epithelial Tcf4 expression was mainly detected in the putative enteric plexus cells (Figure 2A). To analyze Tcf4-deficient mice, we intercrossed *Tcf4^lacZ/+^* mice; however, no viable offspring of the *Tcf4^lacZ/lacZ^* genotype were obtained. Therefore, we performed time pregnancies followed by an analysis of embryos at different developmental stages. The analysis revealed the absence of proliferating cells in the intervillus regions of the small intestine, starting at E16.5 (Figure S1). The phenotype—similar to that seen in *Tcf4^Hyg/Hyg^* or *PGK-Cre Tcf4^floxHMG/floxHMG^* mice—indicated the possible contribution of Tcf4-expressing non-epithelial cells to the formation of intestinal tissue. Nevertheless, the experimental proof (e.g., usage of a Cre driver active in intestinal mesenchymal cells) confirming the hypothesis will require additional experiments.

We never detected any hyperproliferation of progenitor cells in the small intestinal and colonic epithelium as described by Angus-Hill and colleagues [22]. It has been suggested that an N-terminal Tcf4 protein fragment that excludes the DNA binding domain (the HMG box) is expressed from the *Tcf4^Hyg^* (and by analogy from *Tcf4^floxHMG^*) allele and that the protein interferes with TCF/β-catenin-mediated transcription, thus enforcing the phenotype of the Tcf4 knockout mice [40]. Removal of Tcf4 exon 5 results in a frameshift, leading to the production of a (relatively short) polypeptide that is 189 amino acids long. Thus, we tested the possible repressive activity of the polypeptide in a luciferase-based reporter assay. Nevertheless, (over)expression of the N-terminal Tcf4 fragment had no effect on the TCF/β-catenin-dependent transcription (Figure S2). Recently, Vacik and co-workers [41] discovered that during embryonic development, a shortened version of Tcf4 protein missing the β-catenin binding domain is produced. The variant contains the entire C-terminal portion of the protein, including the HMG box. Consequently, it acts as a dominant negative (dn) Tcf4 isoform blocking Wnt-dependent transcription. Additionally, the production of mRNA encoding the dnTcf4 variant is driven by an intronic promoter located upstream of exon 5 [41]. Although the specific Tcf4 isoform was predominantly detected in the developing nervous system, the analogous TCF4 isoform was identified using “in silico” analysis in various human tissues, including the intestine [18]. We suggest that Cre-mediated recombination of the first exon in *Tcf4^flox1/flox1^* mice retains (in contrast to other modified *Tcf4* alleles) the expression of dnTcf4, generating the imbalance between the full-length and the dnTcf4 protein. What cause the difference between Tcf4-deficient cells and cells retaining expression of the dnTcf4 form? Obviously, dnTcf4 represses the transcription of Wnt-signaling target genes [34,42,43]. Interestingly, using a chromatin immunoprecipitation (ChIP)-sequencing (ChIP-seq) experiment, Schuijers and colleagues showed that in comparison to full-length TCF4, dnTCF4 preferentially targets DNA elements bound by other LEF/TCF family members [43]. Consequently, the expression profile of Tcf4 null cells and cells expressing the dnTcf4 isoform would differ. Nevertheless, why the dnTcf4-mediated repression contributes to hyperproliferation of the embryonic intestinal epithelium remains unclear.

### 3.3. Absence of Tcf4 Compromised Cell Proliferation of the Adult Small Intestinal and Colon Epithelia

In young (2-week-old) mice, a slight (nevertheless significant) decrease in the number of crypts and villi was detected in the small intestine of *Tcf4^flox5/flox5^ Villin-Cre* mice (compared to animals with intact Tcf4). In contrast to adult tissue, Tcf4 was mainly present in the crypt epithelium, and not in the cells lining the villi. Tcf4-specific staining was seen not only in wild-type (wt) mice, but also in the crypts of 2-week-old *Tcf4^flox5/flox5^ Villin-Cre* animals, indicating incomplete recombination of the floxed sequences. With respect to the crypt number, the small intestinal epithelium recovered in 10- and 25-week-old mice (Figures S3 and S4). In addition, in the adult animals, the Tcf4-positive cells were located mainly on the villi, and Tcf4 staining in the crypts was less prominent. Interestingly, streams of cells producing Tcf4 were also visible on the villi of *Tcf4^flox5/flox5^ Villin-Cre* mice, confirming incomplete recombination of the *Tcf4* floxed alleles. The latter observation also indicated a strong selection pressure to maintain the Tcf4 expression in epithelial cells. 

**Figure 3:**
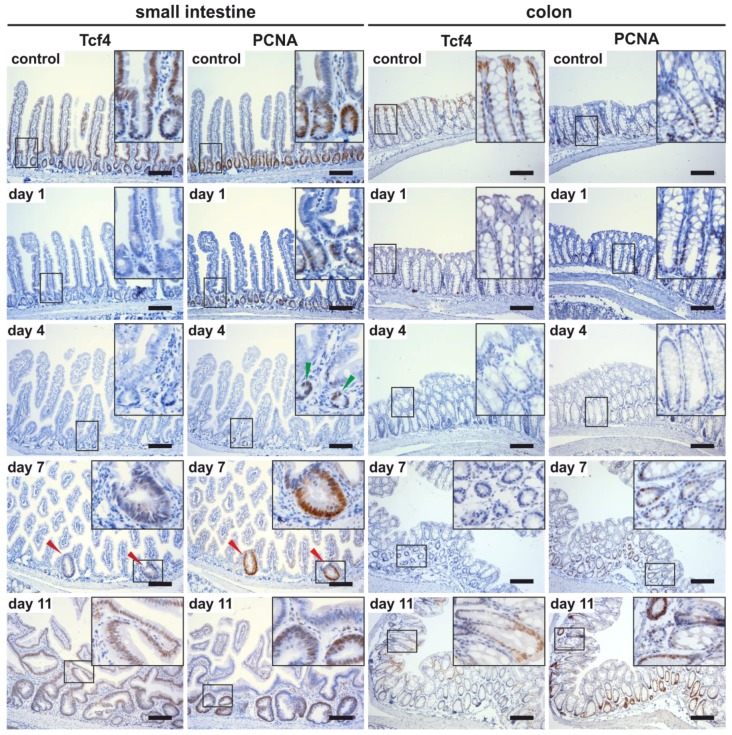
Absence of proliferating crypt cells in the adult Tcf4-deficient intestine. Immunodetection of Tcf4 and PCNA protein in the small intestine and colon of *Tcf4^flox5/flox5^ Villin-CreERT2* mice at indicated time points after tamoxifen administration. Notice the presence of a small number of residual crypts containing PCNA-positive proliferating cells in the small intestine at day 4 after tamoxifen administration (green arrowheads); at day 7, hyperplastic crypts expressing Tcf4 and PCNA are formed (red arrowheads). In the colon epithelium, Tcf4- and PCNA-positive staining is partially restored at day 11. Specimens were counterstained with hematoxylin. Magnified images are shown in the insets. Scale bar: 0.3 mm.

In the colon of 2- and 10-week-old mice, the Tcf4 absence resulted in disorganized epithelia containing patches of normal tissue that retained strong nuclear Tcf4 staining in the cells covering the colon surface. Similarly to the small intestine, partial recovery of the epithelium was observed in 25-week-old mice. Nevertheless, in contrast to the small intestine, the majority of colon tissue remained without Tcf4 expression (Figure S5). The reduced dependency of adult colon tissue on Tcf4 expression in adult mice prompted us to test whether Tcf4 is redundant with other TCFs. However, qRT-PCR or IHC analysis did not show increased expression of any additional LEF/TCF family members in the *Tcf4^flox5/flox5^ Villin-Cre* colon (data not shown). Interestingly, no signs of necrotic death of epithelial cells in the colon—reported after deletion of the *Tcf4^flox1^* allele [22]—were observed. The effect of *Tcf4* deletion in the adult intestinal epithelium was also tested in *Tcf4^flox5/flox5^ Villin-CreERT2* mice. The modified tamoxifen-sensitive ligand binding domain of the estrogen receptor (ERT2) fused to the Cre enzyme allows for excision of the floxed sequences in a timely manner [44]. Experimental animals were sacrificed 1, 4, 7, and 11 days after tamoxifen administration, i.e., *Tcf4* gene inactivation. Immunohistochemical staining revealed the absence of Tcf4 protein in the small intestinal and colon samples as early as one day after tamoxifen administration. Tcf4 absence was accompanied by a loss of proliferating cells in the crypts of both tissues. At day 7, several vigorously proliferating hyperplastic crypts expressing Tcf4 were observed in the small intestine. These crypts at day 11 expanded, forming disorganized epithelium with distorted morphology (Figure 3). The colon architecture was—besides the absence of PCNA-positive cells—seemingly not affected. However, fluorescence-activated cell sorting (FACS) analysis showed reduced proportion of cells expressing CD24, indicating that the tissue underwent cellular (and functional) changes (Figure S6). In the colon, proliferating cells in the crypts re-appeared at day 7 and, simultaneously, nuclear Tcf4-specific staining was observed in some crypt cells. At day 11, Tcf4 production was clearly visible in differentiated cells on the tissue surface (Figure 3). Tcf4-deficient animals had to be sacrificed prior to day 12 because of malnutrition precluding any analysis at later time points. Nevertheless, it was evident that in contrast to *Tcf4^flox5/flox5^ Villin-Cre* mice, cells in which the *Tcf4* gene escaped “acute” inactivation were the source of epithelial recovery in the *Tcf4^flox5/flox5^ Villin-CreERT2* colon.

### 3.4. Tcf4-Deficient Intestinal Stem Cells s Do Not Contribute to Intestinal Homeostasis

**Figure 4:**
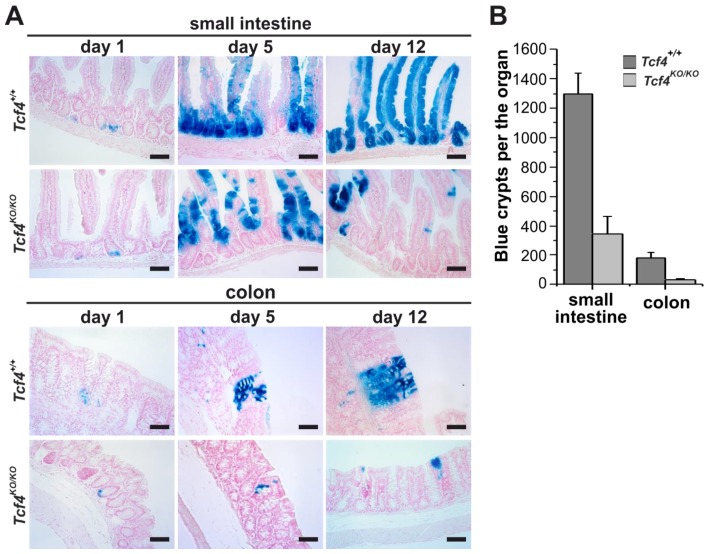
Depletion of the *Tcf4*-deficient cell clones from the intestinal epithelium. (**A**) Lineage tracing in the small intestine and colon of *Tcf4^+/+^ Rosa26R-lacZ Lgr5-CreERT2* (*Tcf4^+/+^*) and *Tcf4^flox5/flox5^ Rosa26R-lacZ Lgr5-CreERT2* (*Tcf4^KO/KO^*) mice at indicated time points after tamoxifen administration. The tissue sections were stained with X-gal to visualize the lacZ-expressing cells (blue color). Specimens were counterstained with nuclear fast red. Scale bar: 0.15 mm; (**B**) Quantification of labeled crypts present in the small intestinal and colonic epithelium 12 days after tamoxifen administration. Four animals of each genotype were analyzed; all blue crypts in the middle portion of the small intestine and colon were counted; error bars indicate SDs. Scale bar: 0.15 mm.

Subsequently, we employed *Rosa26R-lacZ* reporter mice to trace the fate of ISCs upon Tcf4 ablation. *Rosa26R-lacZ* animals harbor the *lacZ* gene integrated downstream of the *Rosa26* promoter. However, although the *Rosa26* locus is ubiquitously active, *lacZ* mRNA is produced only after Cre-mediated removal of the floxed transcriptional blocker placed upstream of the *lacZ* gene [45]. Recombination of the floxed sequences was induced in adult *Tcf4^flox5/flox5^ Rosa26R-lacZ Lgr5-CreERT2* and control *Tcf4^+/+^ Rosa26R-lacZ Lgr5-CreERT2* animals by a single dose of tamoxifen. LacZ staining was followed at several points after tamoxifen administration. At day 1, lacZ-positive cells were observed at the bottom of the small intestinal and colonic crypts in both mouse strains. At day 5, streams of blue cells expanded from the crypts in animals of both genotypes. However, in Tcf4-deificient epithelium, the streams frequently separated from the crypt bottoms. Twelve days after recombination, continuous “ribbons” of labelled cells emanating from the crypts and reaching the top of the villi or colonic surface were observed in control mice. However, in Tcf4-deficient animals, the majority of blue cells disappeared from the intestine (Figure 4A). Subsequent quantification confirmed substantially reduced numbers of persistently labelled crypts in Tcf4-deficient animals when compared to mice with the wt *Tcf4* alleles (Figure 4B).

### 3.5. Tcf4 Loss Affects the Size of Apc-Deficient Small Intestinal Tumors

**Figure 5:**
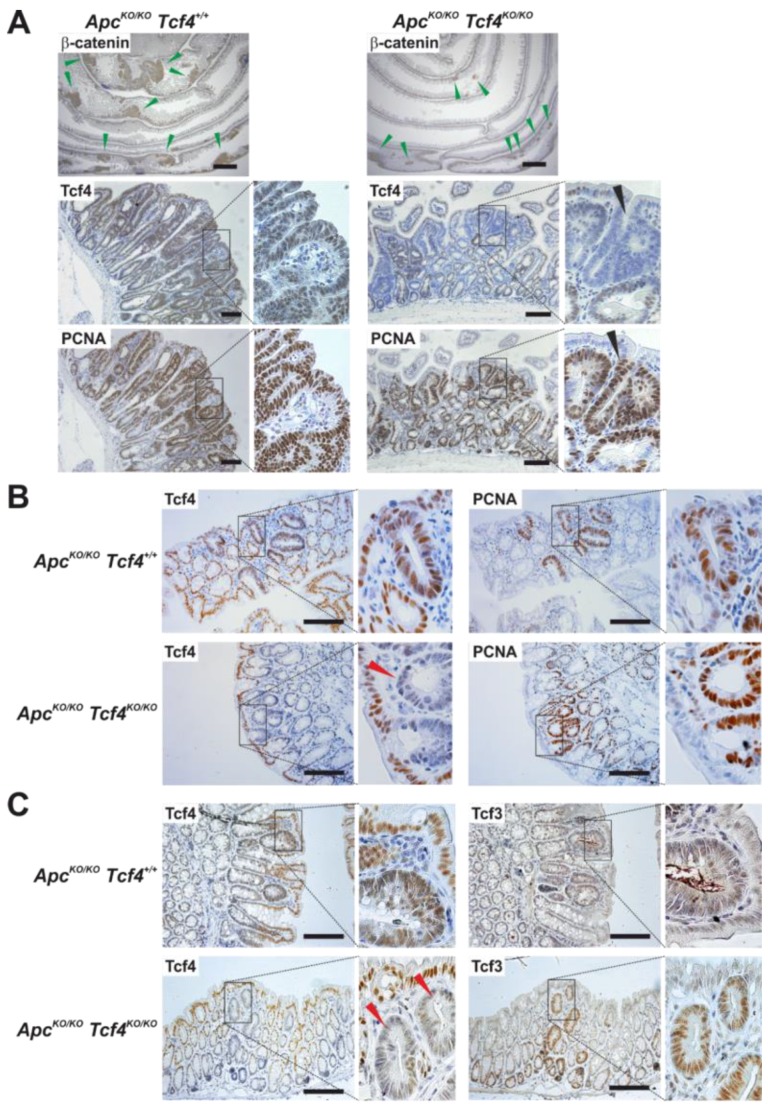
Tcf4 inactivation reduces tumor formation in the small intestine. Immunohistochemical staining of the small intestinal (**A**) and colon (**B**) tumors generated in *Apc^flox14/flox14^ Tcf4^+/+^ Lgr5-CreERT2* (*Apc^KO/KO^ Tcf4^+/+^*) and *Apc^flox14/flox14^ Tcf4^flox5/flox5^ Lgr5-CreERT2* (*Apc^KO/KO^ Tcf4^KO/KO^*) mice 28 days after tamoxifen administration. Notice the reduced size of lesions in Tcf4-deficient small intestine compared to mice with the intact Tcf4 (green arrowheads); (**C**) Increased expression of Tcf3 in the colonic tumors of *Apc^KO/KO^ Tcf4^KO/KO^* mice 28 days after tamoxifen administration. Some small intestinal tumors are without Tcf4 staining; however, they are PCNA-positive (black arrowhead). In contrast, colonic lesions retain residual Tcf4 positivity (red arrowheads). Specimens were counterstained with hematoxylin; magnified images are shown in the insets. Scale bar: 0.3 mm.

To elucidate the role of Tcf4 in intestinal tumorigenesis, we used mice with the conditional *Apc* allele containing exon 14 flanked by *loxP* sites (*Apc^flox14/flox14^*). The Cre-mediated excision of the floxed exon changes the reading frame downstream of the deletion and leads to the production of a truncated, non-functional Apc polypeptide [46]. Animals harboring the floxed or wt *Tcf4* alleles, i.e., *Apc^flox14/flox14^ Tcf4^flox5/flox5^ Lgr5-CreERT2* or *Apc^flox14/flox14^ Tcf4^+/+^ Lgr5-CreERT2* mice, respectively, were treated with a reduced dose of tamoxifen (1 mg per animal) to increase their survival. In the small intestine, concomitant inactivation of Tcf4 and Apc resulted in a significant decrease of the size of neoplastic lesions. Interestingly, IHC analysis showed that in some PCNA-positive, i.e., proliferating tumor parts, Tcf4-specific staining was absent, indicating that the proliferation of transformed cells is Tcf4-independent (Figure 5A). Due to the less efficient Lgr5-CreERT2-mediated removal of the floxed sequences [7,47], less abundant neoplastic lesions were formed in the colon. Nevertheless, Tcf4 inactivation had no effect on the size and amounts of colonic (micro)adenomas (Figure 5B). Moreover, in contrast to the small intestine, all colonic lesions retained Tcf4-specific residual staining, suggesting incomplete Tcf4 inactivation. Moreover, we detected increased expression of Tcf3 in neoplastic colon tissue, suggesting that the decreased dosage of Tcf4 was compensated for by increased Tcf3 production (Figure 5C). Since Tcf3 expression was never linked to (hyper)active Wnt signaling, a mechanistic explanation for its elevated production in Apc-deficient neoplastic lesions is unclear. Contrary to our results, Angus-Hill and co-workers found that increased expression of additional LEF/TCF family members Lef1 and Tcf1 in colon tumors of Min mice harboring only one intact *Tcf4* allele (in comparison to tumors with both alleles functional). In the same study, no significant change in Tcf3 expression was observed [22]. We suggest that in larger, more progressed tumors, the reduced dosage of Tcf4 activity might be compensated for by Tcf1 and/or Lef1, whereas in early lesions, the Tcf4 role is substituted by Tcf3.

### 3.6. Tcf4-Deficient Organoids Displayed Impaired Growth

**Figure 6:**
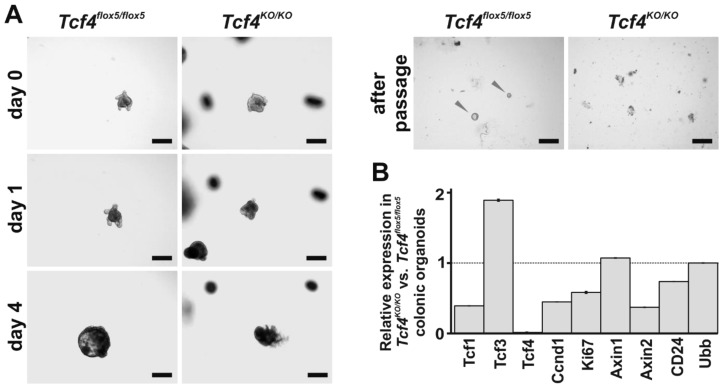
Impaired growth of colonic organoids after *Tcf4* gene inactivation. (**A**) Stereomicroscopic images of organoids derived from the *Tcf4^flox5/flox5^* and *Tcf4^flox5/flox5^ Villin-CreERT2* (*Tcf4^KO/KO^*) colon. The images were taken prior to (day 0), one, and four days after 4-hydroxytamoxifen (4-OHT) treatment. The images at the right show organoids one day after splitting. Scale bar: 200 μm; (**B**) qRT-PCR analysis of total RNA isolated from *Tcf4^flox5/flox5^* and *Tcf4^KO/KO^* colonic organoids three days after 4-OHT treatment. Control samples were obtained from organoid cultures treated with solvent (ethanol). RNA samples were isolated from three different organoid cultures established from mice of the corresponding genotype. Results were normalized to the *Actb* mRNA levels; *Ubiquitin B* (*Ubb*) represents an additional housekeeping gene. Gene expression levels in ethanol-treated organoids were set to 1. Error bars indicate SDs. Notice that *Lef1* expression was not detected; the *Ccnd1* gene encodes cyclin D1 cell cycle regulator.

To address whether Tcf4 absence affects the growth of intestinal epithelial cells in vitro, organoid cultures were established from the crypts explanted from the small intestine and colon of *Tcf4^flox5/flox5^ Villin-CreERT2* mice; control organoids were derived from *Tcf4^flox5/flox5^* animals. Recombination of the floxed sequences was induced by 4-OHT, and the organoid growth and morphology was monitored. In accordance with data published previously [20], Tcf4-deficient organoids grew comparably to *Tcf4^flox5/flox5^* organoids until day 4 after 4-OHT addition to the culture medium. Since day 4, Tcf4-deficient organoids showed intermitted budding, and started to release dead cells from the lumen. Additionally, the organoids were unable to restart their growth after a single passage (Figure 6A and Figure S7A). qRT-PCR analysis of total RNA isolated from organoids three days after 4-OHT treatment showed a clear decrease in the level of *Tcf4* mRNA in CreERT2-expressing organoids. This was accompanied by robust downregulation of proliferating cell markers (*Ki67*, *cyclin D1*) and Wnt signaling responsive genes (*Axin2*, *Lef1*, *Tcf1*) [48,49,50]. The expression levels of *Axin2* paralog *Axin1*, i.e., the gene that is not regulated by the Wnt pathway, did not differ between Tcf4-deficient and control samples. Interestingly, *Tcf3* expression was two-fold upregulated in colon organoids after Tcf4 inactivation. Moreover, in agreement with the FACS analysis, Tcf4 ablation decreased production of CD24 in colon organoids (Figure 6B and Figure S7B).

**Figure 7:**
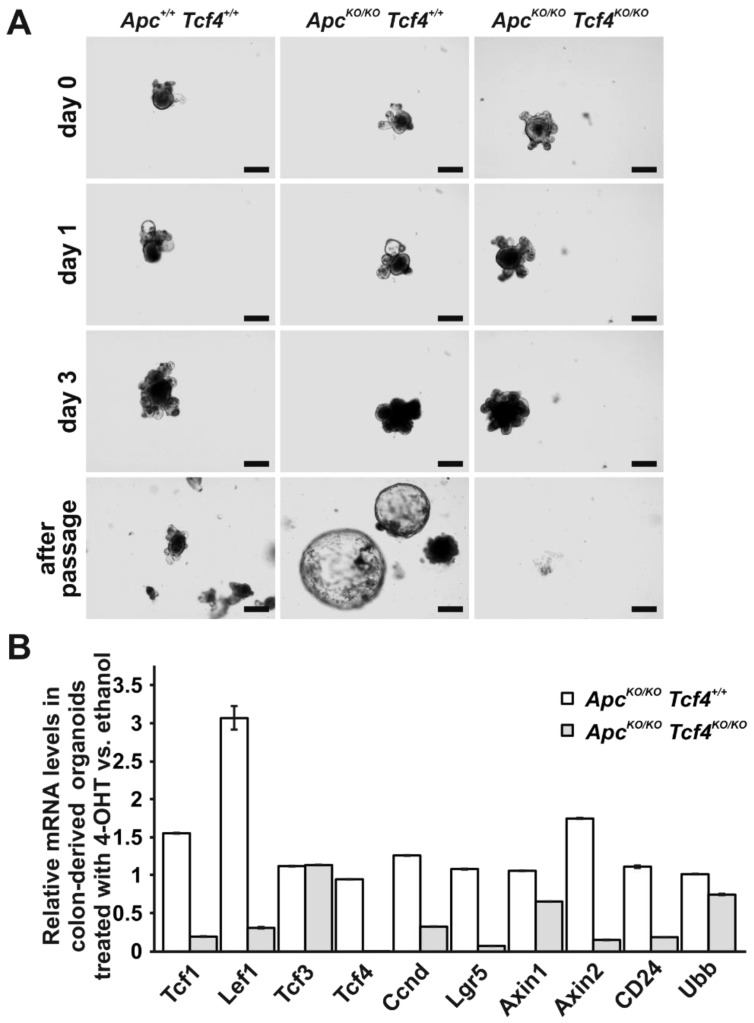
Demise of Apc/Tcf4 double-deficient colonic organoids. (**A**) Stereomicroscopic images of organoids derived from the colon of *Apc^+/+^ Tcf4^+/+^ Lgr5-CreERT2* (*Apc^+/+^ Tcf4^+/+^*), *Apc^flox14/flox14^ Tcf4^+/+^ Lgr5-CreERT2* (*Tcf4^+/+^ Apc^KO/KO^*), and *Apc^flox14/flox14^ Tcf4^flox5/flox5^ Lgr5-CreERT2* (*Apc^KO/KO^ Tcf4^KO/KO^*) mice. The images were taken prior to (day 0), one and four days after 4-OHT treatment. The images at the bottom show organoids one day after splitting. Scale bar: 200 μm; (**B**) qRT-PCR analysis of organoids derived from the indicated mouse strains. Total RNA was isolated from four (parallel) organoid cultures 48 h after 4-OHT or ethanol addition to culture media. Diagrams show the relative expression levels of the indicated genes in ethanol-treated organoids when compared to 4-OHT-treated organoids of the corresponding genotype. RNA levels were normalized to the *Actb* mRNA levels; *Ubb* represents an additional housekeeping gene. Error bars indicate SDs.

**Figure 8:**
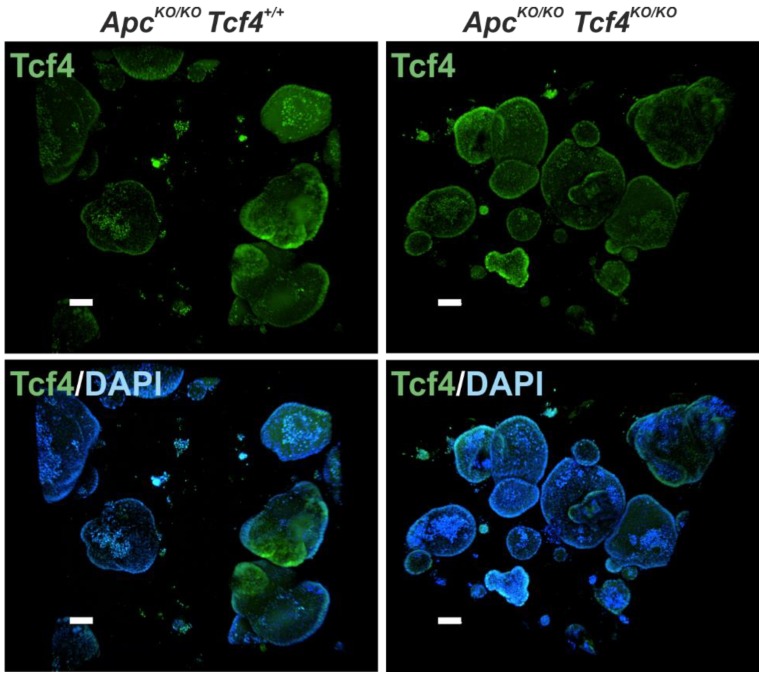
Small intestinal organoids derived from the transformed epithelium produce Tcf4. Fluorescent microscopy images of tumor organoids (spheroids) derived from the small intestine of *Apc^flox14/flox14^ Tcf4^+/+^ Lgr5-CreERT2* (*Apc^KO/KO^ Tcf4^+/+^*) and *Apc^flox14/flox14^ Tcf4^flox5/flox5^ Lgr5-CreERT2* (*Apc^KO/KO^ Tcf4^KO/KO^*) mice 28 days after tamoxifen administration. Samples were stained using an anti-TCF4 antibody (green fluorescence) and they were counterstained using diamidino-2-phenylindole (DAPI) stain (blue nuclear fluorescence). Scale bar: 100 μm.

Next, we derived small intestinal or colon organoids from *Apc^flox14/flox14^ Tcf4^flox5/flox5^ Lgr5-CreERT2*, *Apc^flox14/flox14^ Tcf4^+/+^ Lgr5-CreERT2*, and *Apc^+/+^ Tcf4^+/+^ Lgr5-CreERT2* mice, and induced single Apc or double Apc/Tcf4 inactivation by 4-OHT. Apc inactivation in organoids harboring the unmodified *Tcf4* gene led to the formation of fast growing “spheroids” displaying a cyst-like morphology lacking the budding crypt domains (the morphological change was mainly evident after organoid passage). However, simultaneous deletion of Apc and Tcf4 caused organoid demise after organoid culture splitting (Figure 7A and data not shown). Subsequent qRT-PCR analysis revealed that the Apc inactivation caused robust upregulation of *Lef1* mRNA and a moderate increase in the expression levels of other Wnt signaling target genes *Axin2*, *Tcf1*, and *cyclin D1* in both colonic and small intestinal organoids. Additionally, Apc-deficient small intestinal organoids displayed increased levels of *Tcf3* mRNA. However, concomitant deletion of Apc and Tcf4 led to a substantially diminished expression of all tested Wnt target genes, including *Axin2*, *Lef1*, *Lgr5*, and, *Tcf1*, and crypt base cells marker *CD24* (Figure 7B and Figure S8). Finally, we derived organoids from the hyperplastic small intestine of *Apc^flox14/flox14^ Tcf4^flox5/flox5^ Lgr5-CreERT2* mice treated with tamoxifen. As the neoplastic tissue contained Tcf4-negative tumor cells (Figure 5A), we hypothesized that in growing tumors, some transformed ISCs might lose their dependency on Tcf4. Control organoids were established from tamoxifen-treated *Apc^flox14/flox14^ Tcf4^+/+^ Lgr5-CreERT2* mice, and the Tcf4 protein was visualized in both types of organoids (that were growing as typical tumor spheroids) using fluorescent microscopy. Nevertheless, tumor organoids derived from both mouse strains produced similar levels of Tcf4 (Figure 8). This showed that Tcf4 is indispensable for organoid establishment or growth, even in the absence of Apc.

### 3.7. Redundancy of LEF/TCF Family Members in Human APC-Deficient Cells

**Figure 9:**
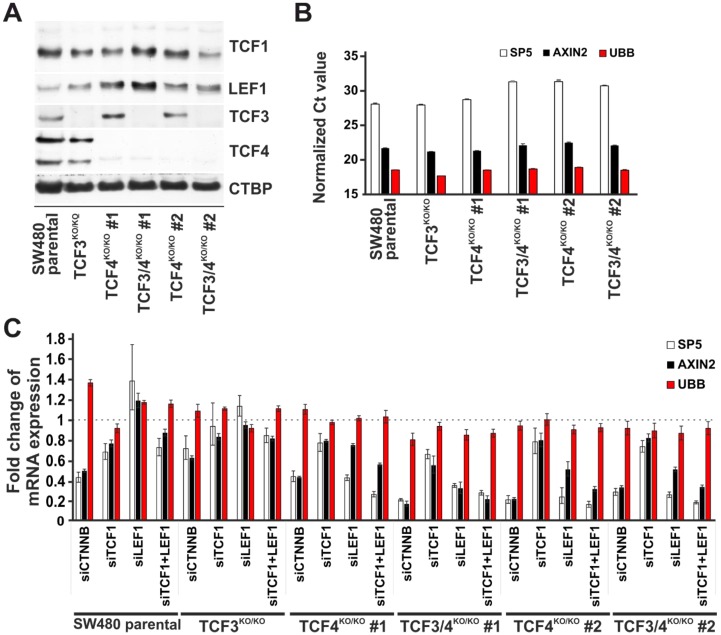
TCF4 is dispensable for TCF/β-catenin-dependent transcription in SW480 cancer cells. (**A**) Western blot analysis of whole-cell lysates prepared from parental SW480 cells or TCF3/TCF4 single- or double-deficient cell clones generated by CRISPR/Cas9 targeting. CTBP, control immunoblotting with C-terminal Binding Protein (CTBP)-specific antibody. (**B**) qRT-PCR analysis of Wnt signaling target genes *AXIN2* and *SP5* in indicated cell clones. The diagram shows Ct values upon normalization to *ACTB* expression. (**C**) qRT-PCR analysis of *AXIN2* and *SP5* in the indicated cells upon siRNA-mediated knockdown of TCF1 and/or LEF1 expression. CTNNB-specific (the gene encodes β-CATENIN) small interfering RNA (siRNA) was used to monitor the sensitivity of the selected genes to Wnt pathway downregulation. RNA levels were normalized to *ACTB* expression; *UBB* represents an additional housekeeping gene; the expression level of a given gene in the cells treated with control non-silencing siRNA (siCTRL) was set to 1. The experiments in (**B**,**C**) were performed twice (each experiment in triplicates); representative results are shown. *UBB* represents an additional housekeeping gene; error bars indicate SDs.

Active Wnt signaling represents a hallmark of the majority of CRC [16]. In addition, initial studies indicated that TCF4 is the major mediator of aberrant Wnt signaling in CRC cells [11,14]. Our recent analysis of various gene expression databases indeed confirmed that *TCF4* displays—among the LEF/TCF family members—the highest expression in the human intestine and colon. Nevertheless, the same analysis also showed that besides TCF4, all other TCFs are expressed in healthy or tumor intestinal tissue [18]. The latter finding was verified experimentally by immunoblotting, which showed that LEF/TCF family members are produced in APC-deficient SW480 CRC cells and M1 cells (Figure 9A and Figure S9A). M1 cells were generated from parental HEK293 by transcription activator-like effector nucleases (TALEN)-mediated targeting of the *APC* locus. Consequently, the cells produce a truncated form of APC, leading the constitutive Wnt pathway activity, thus mimicking the situation in the majority of CRC. To test the TCF4 contribution to TCF/β-catenin-dependent transcription, we employed the CRISPR/Cas9 system to disrupt *TCF4* and/or its closest paralog *TCF3*. We generated TCF3/TCF4 single- or double-deficient SW480 and M1 single cell clones. The clones were viable and did not change their proliferation rate when compared to the parental cells (Figure S10). Total RNA isolated from several cell clones obtained from each gene targeting experiment was used to analyze the expression levels of Wnt-responsive genes *AXIN2* [48,51] and *SP5* [52]. Representative results are summarized in Figure 9B and Figure S9B. In SW480 cells, the TCF3 absence had no effect on the levels of *AXIN2* and *SP5* mRNA. Moreover, in single TCF4 knockout cells, we observed either a negligible effect or a slight reduction in the expression levels of both tested genes. Additional disruption of *TCF3* in TCF4-deficient cells either had no additive effect, or it further potentiated TCF4 loss (Figure 9B). In M1 cells, single or double disruption of TCF3 and/or TCF4 had virtually no effect on *AXIN*2 and *SP5* expression (Figure S9B). Since the obtained results implied redundancy of TCF3/4 proteins with LEF1 and/or TCF1, we used RNAi to downregulate the production of the latter factors. As shown in Figure 9C and Figure S9C, the most robust decrease in *AXIN2* and *SP5* expression levels was observed in single TCF4-deficient cells treated simultaneously with siRNA against LEF1- and TCF1-specific siRNAs. TCF3/TCF4 double deficiency had—depending on the individual clone analyzed—either no additional effect, or it further decreased the mRNA levels of the analyzed Wnt target genes. In summary, the results showed mutual interchangeability among TCFs in human cells. Moreover, although we cannot exclude the possibility that different LEF/TCF family members regulate different sets of target genes, we did not observe any transcriptional repression activity of the TCF4 (or TCF3) protein.

## 4. Conclusions

TCF4 is the major nuclear mediator of canonical Wnt signaling in the mouse intestine and human colorectal cancer cells. However, several groups have reported discrepant results related to the TCF4 function in the embryonic or adult mouse gut tissue. Moreover, recent genetic analysis of human tumor specimens indicated a possible tumor suppressive role of TCF4 in a significant fraction of colorectal carcinomas. We employed a newly generated floxed *Tcf4* allele that allows inactivation of all potential Tcf4 isoforms produced in the mouse tissues. The allele was combined with several intestinal-specific Cre drivers to perform continuous or timed Tcf4 gene ablation in intestinal stem cells or throughout the epithelium of the small intestine and colon. Additionally, we utilized the CRISPR/Cas9 system to disrupt the *TCF4* gene and its closes homolog *TCF3* in two human cell lines and quantified the impact of the disruption on expression of Wnt signaling target genes *AXIN2* and *SP5*.

Targeted deletion of Tcf4 in the adult gut was accompanied by a loss of proliferating cells in both the small intestine and colon. Moreover, lineage tracing experiments showed that adult Tcf4-deficient small intestinal and colon stem cells do not contribute to epithelial self-renewal. During embryogenesis, epithelial expression of the *Tcf4* gene was seemingly less essential. The absence of any (strong) phenotype in the Tcf4-deficient developing gut might be caused by incomplete recombination of the floxed allele and/or by direct involvement of non-epithelial Tcf4-expressing cells in the intestinal epithelium formation.

We did not observe a tumor suppressive effect of Tcf4. In fact, concomitant deletion of Tcf4 and Apc resulted in a significant decrease in the size of small intestinal tumors. Moreover, all colonic lesions retained residual Tcf4 expression. Additionally, Tcf4 appeared essential for the growth of small intestinal or colon organoids irrespective of the Apc status. The Tcf4 necessity was mainly manifested during expansion of the organoid cultures. Contrary to the results obtained in the mouse, TCF4 (and TCF3) knockout in APC-deficient human cells had no remarkable effect on cell growth or transcription of the Wnt signaling target genes. Subsequent siRNA experiments confirmed the redundancy of TCF4 with LEF1 and TCF1.

In summary, our results showed the importance of the Tcf4 Wnt effector, mainly in the mouse model of adult intestinal epithelium homeostasis and tumor initiation. In human cells, other TCF/LEF family members substitute for the TCF4 role, probably due to different “wiring” of the intracellular signaling mechanism.

## Supplementary Materials

The following materials are available online at http://www.mdpi.com/2073-4425/9/9/439/s1. Figure S1: Impaired proliferation of the Tcf4-deficient small intestine; Figure S2: The N-terminal portion of TCF4 does not interfere with β-catenin/TCF-dependent transcription; Figure S3: Immunohistochemical analysis of the middle portion of the small intestine of *Tcf4^flox5/flox5^* and *Tcf4^flox5/flox5^ Villin-Cre* (*Tcf4^KO/KO^*) mice of the indicated age; Figure S4: Reduced numbers of the crypts in the small intestine of 2-week-old *Tcf4^flox5/flox5^ Villin-CreERT2* (*Tcf4^KO/KO^*) mice when compared to *Tcf4^flox5/flox5^* littermates; Figure S5: Continuous inactivation of the *Tcf4* gene impairs postnatal formation of the colon epithelium; Figure S6: Reduced proportion of CD24+ cells in the Tcf4-deficient colon; Figure S7: Impaired growth of small intestinal organoids after *Tcf4* gene inactivation; Figure S8: qRT-PCR analysis of small intestinal organoids derived from *Tcf4^+/+^ Apc^flox14/flox14^ Lgr5-CreERT2* (*Tcf4^+/+^ Apc^KO/KO^*), and *Tcf4^flox5/flox5^ Apc^flox14/flox14^ Lgr5-CreERT2* (*Apc^KO/KO^ Tcf4^KO/KO^*) mice; Figure S9: TCF4 is dispensable for TCF/β-catenin-dependent transcription in M1 human cells; Figure S10: Cell viability test of parental SW480 cells or TCF3/TCF4 single- or double-deficient cell clones. Table S1: Oligonucleotide primers used in the study.
