# Supplementary material for: Wnt Effector TCF4 Is Dispensable for Wnt Signaling in Human Cancer Cells
Source: Genes (Basel). 2018 Sep 1;9(9):439. doi: 10.3390/genes9090439 (PMC6162433; doi:10.3390/genes9090439)
Supplement: Supplementary file 1 [file genes-09-00439-s001.pdf]

genes

MDPI

Supplementary Figure and Table Legends

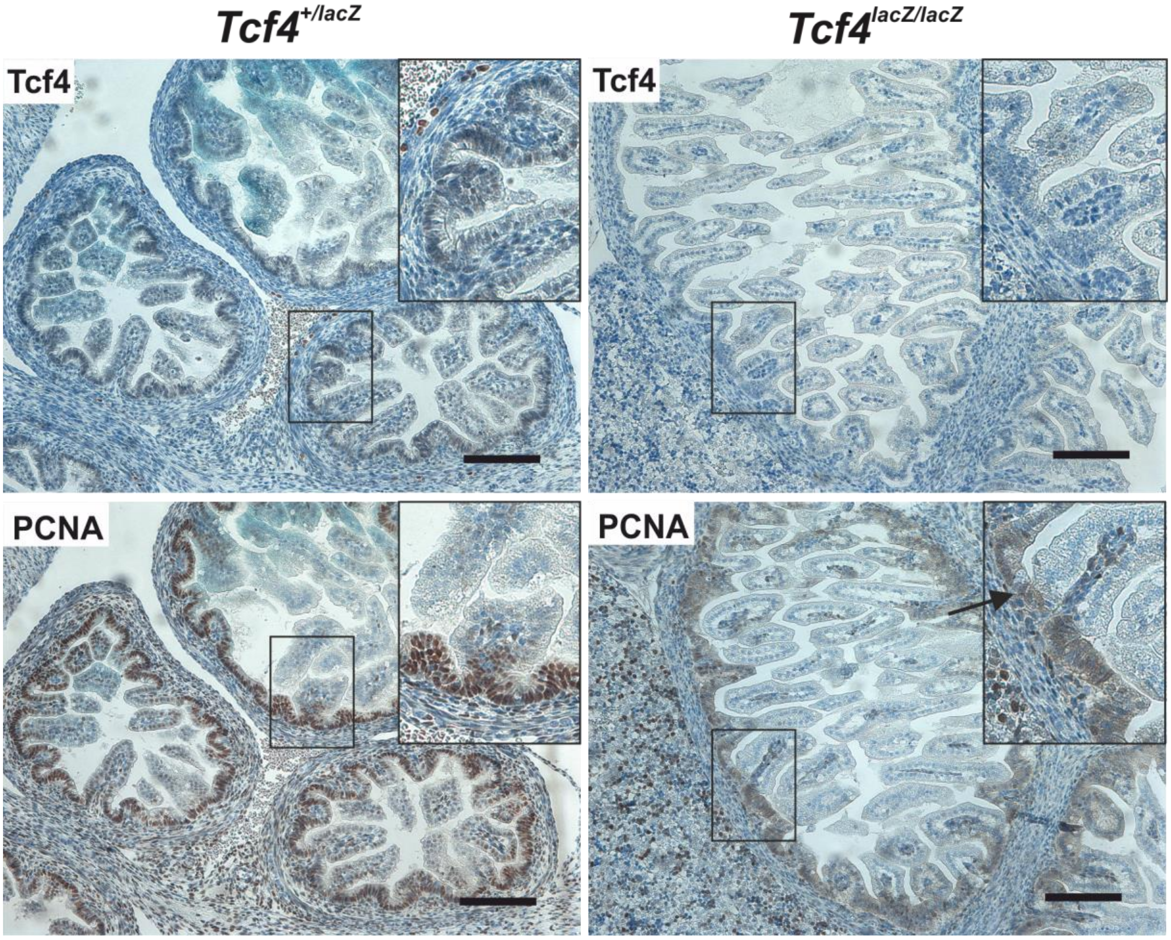

Figure S1. Impaired proliferation of the Tcf4-deficient small intestine. Immunohistochemical analysis of the small intestine of heterozygous (*Tcf4*$^{lacZ/+}$) and Tcf4-deficient (*Tcf4*$^{lacZ/lacZ}$) mice at E17.5. Notice the reduced numbers of cells producing the proliferating cell nuclear antigen (PCNA) in the intervillus regions of *Tcf4*$^{lacZ/lacZ}$ animals (black arrow). Specimens were counterstained with hematoxylin. Magnified images are shown in the insets. Scale bar: 0.3 μm.

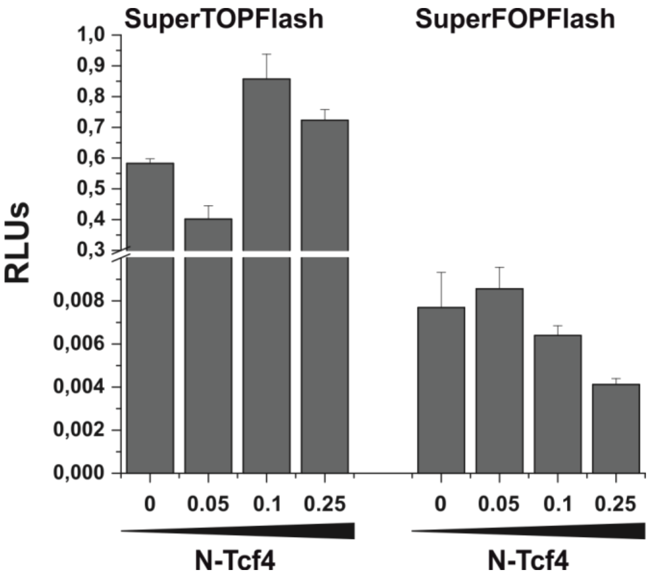

Figure S2. The N-terminal portion of TCF4 does not interfere with TCF/β-catenin-dependent transcription. Luciferase reporter assay in APC-deficient SW480 cells. The cells were transfected with the Wnt reporter SuperTOPFlash (or the mutated variant SuperFOPFlash for control), increasing the amounts of the expression vector encoding the N-terminal portion (amino acids 1-189) of mouse Tcf4 (N-Tcf4), and the Renilla expression plasmid. The diagrams show relative luciferase units (RLUs) normalized to the Renilla activity. The assay was performed in technical triplicates. Error bars indicate SDs.

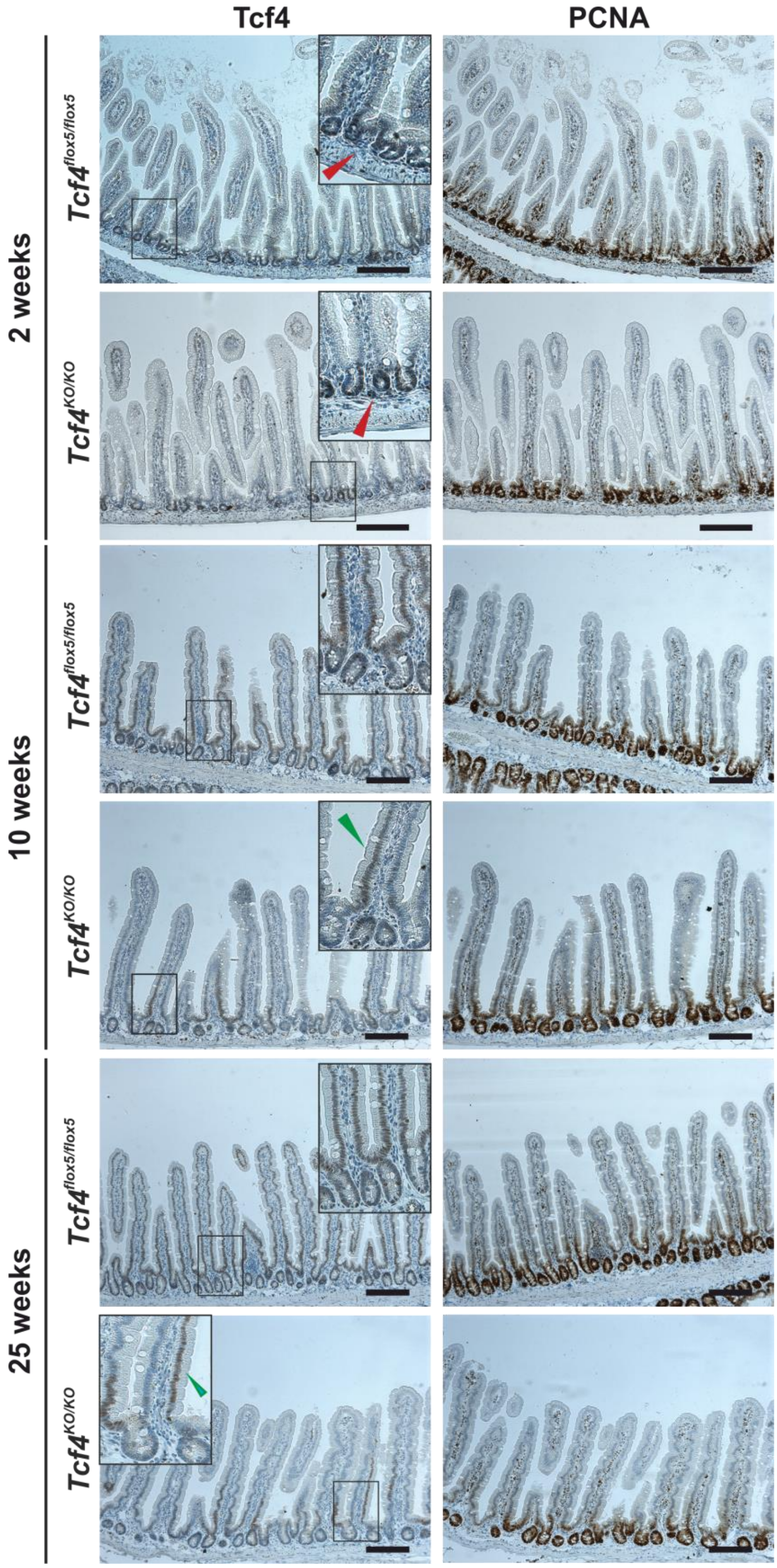

Figure S3. Immunohistochemical analysis of the middle portion of the small intestine of *Tcf4*$^{flox5/flox5}$ and *Tcf4*$^{flox5/flox5}$ *Villin-Cre* (*Tcf4*$^{KO/KO}$) mice of the indicated age. Tcf4-positive cells appeared in the crypts in 2-week-old mice of both genotypes (red arrowheads). In adult *Tcf4*$^{KO/KO}$ animals, stripes of cells producing Tcf4 are visible on the villi (green arrowheads). Specimens were counterstained with hematoxylin. Scale bar: 0.3 mm.

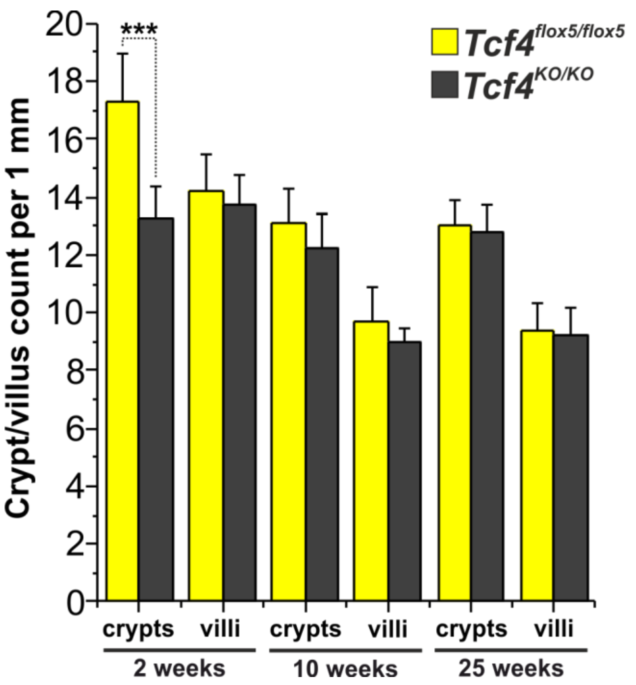

Figure S4. Reduced numbers of the crypts in the small intestine of 2-week-old *Tcf4*$^{flox5/flox5}$ *Villin-CreERT2* (*Tcf4*$^{KO/KO}$) mice when compared to *Tcf4*$^{flox5/flox5}$ littermates. The diagram shows the average number of the crypts or villi per millimeter of the length in the middle portion (jejunum) of the small intestine. Two mice for each indicated age and genotype were analyzed. A total distance of 30 mm of tissue was analyzed per animal; SDs, standard error; ***, $p < 0.001$ (Student's *t*-test).

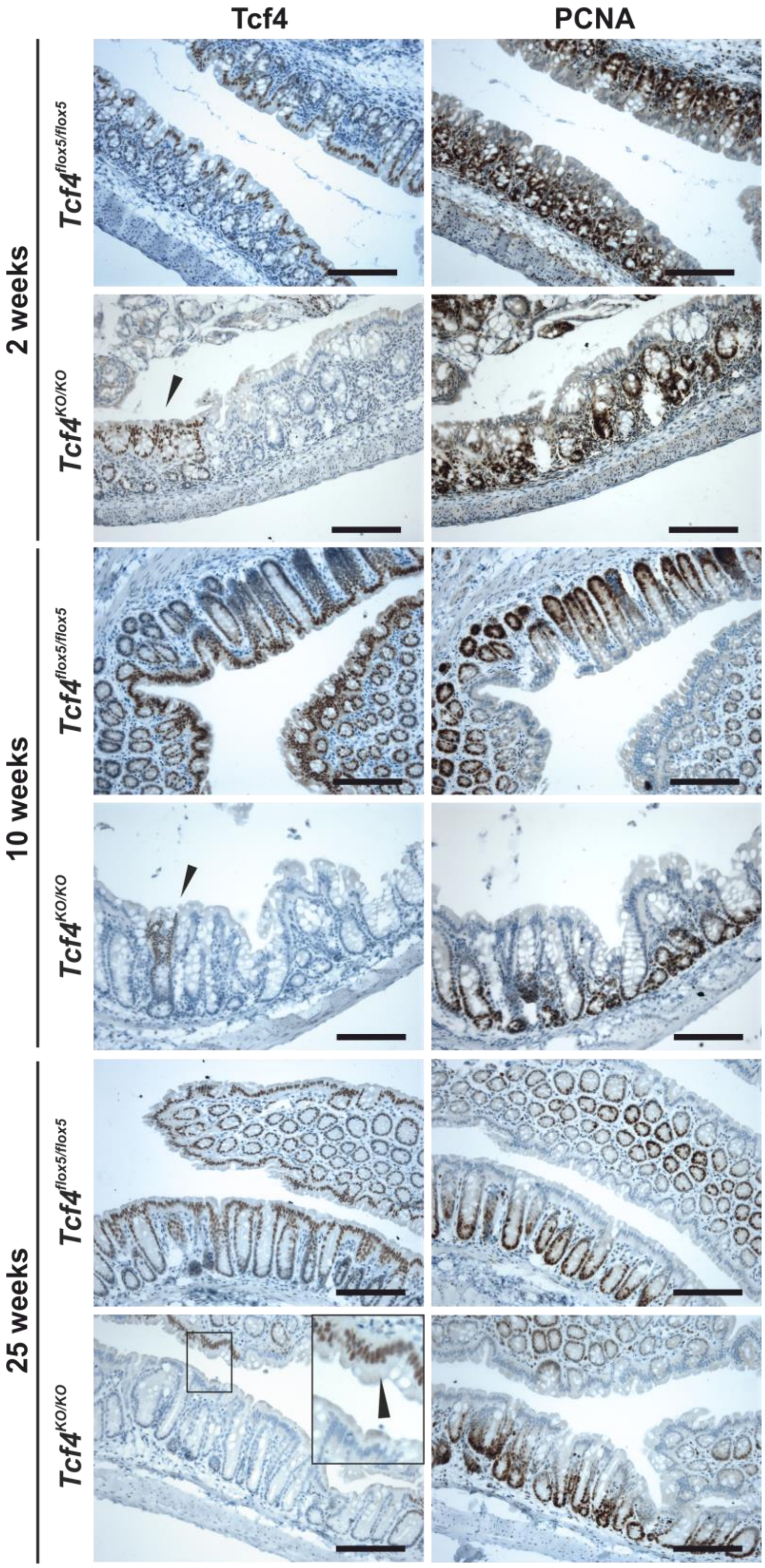

Figure S5. Continuous inactivation of the *Tcf4* gene impairs postnatal formation of the colon epithelium. Immunohistochemical analysis of the middle portion of the colon of $Tcf4^{flox5/flox5}$ and $Tcf4^{flox5/flox5}$ *Villin-Cre* ($Tcf4^{KO/KO}$) mice of the indicated age. Notice the disorganized colonic epithelium in 2- and 10-week-old mice and the normal morphology of the tissue in 25-week-old animals. Black arrowheads point to Tcf4-positive patches. Specimens were counterstained with hematoxylin. Scale bar: 0.3 mm.

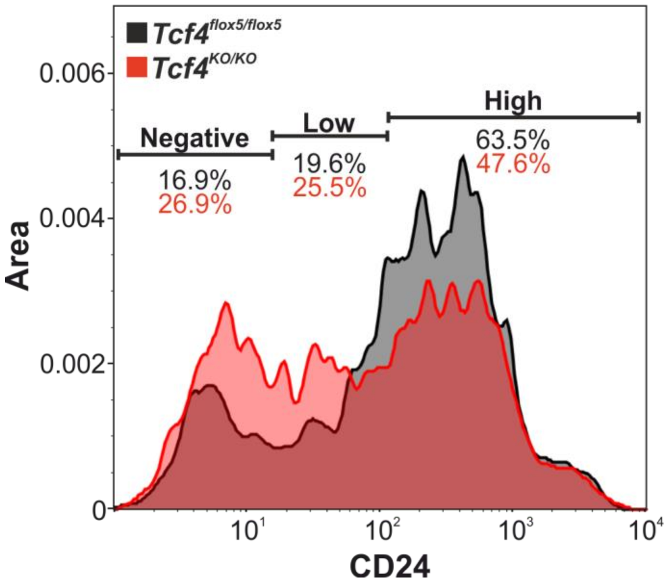

Figure S6. Reduced proportion of CD24⁺ cells in the Tcf4-deficient colon. Fluorescence-activated cell sorting analysis of epithelial cells isolated from *Tcf4$^{flox5/flox5}$ Villin-CreERT2 Rosa26R-tdTomato* (*Tcf4$^{KO/KO}$*) or *Tcf4$^{flox5/flox5}$ Rosa26R-tdTomato* (*Tcf4$^{flox5/flox5}$*) mice four days after a single dose of tamoxifen. Only cells with red fluorescence, i.e. Cre-recombined epithelial cells, were used to quantify CD24-high, CD24-low and CD24-negative cells. Cells were obtained from three mice of each strain, pooled, and analyzed.

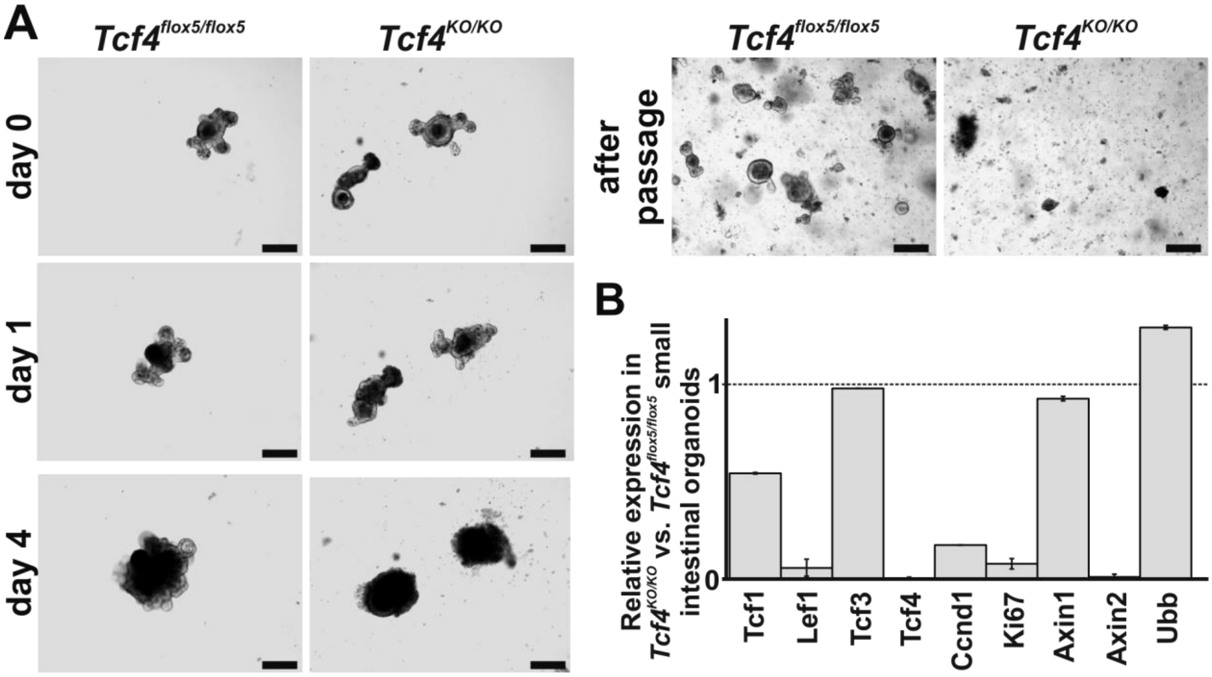

Figure S7. Impaired growth of small intestinal organoids after *Tcf4* gene inactivation. (**A**) Stereomicroscopic images of organoids derived from the *Tcf4*$^{flox5/flox5}$ and *Tcf4*$^{flox5/flox5}$ *Villin-CreERT2* (*Tcf4*$^{KO/KO}$) small intestine. The images were taken prior to, one and four days after 4-hydroxytamoxifen (4-OHT) treatment. The images at the right show organoids one day after splitting. Intact growing organoids are indicated by green arrowheads. Scale bar: 200 μm. (**B**) Quantitative RT-PCR analysis of total RNA isolated from *Tcf4*$^{flox5/flox5}$ and *Tcf4*$^{KO/KO}$ small intestinal organoids three days after 4-OHT treatment. RNA samples were isolated from three different organoid cultures of the corresponding genotype. Results were normalized to the *β-actin* (*Actb*) mRNA levels. *Ubiquitin B* (*Ubb*) represents an additional housekeeping gene. Error bars indicate SDs; the *Ccnd1* gene encodes cyclin D1.

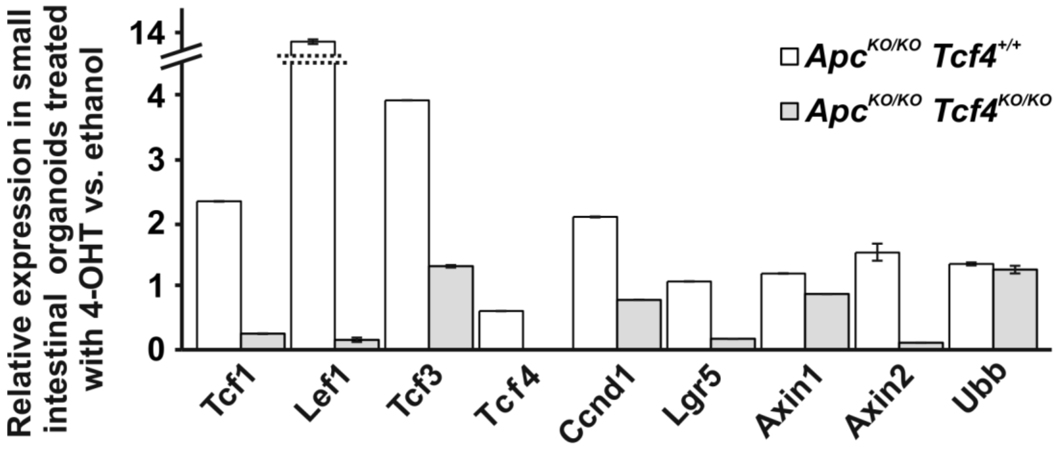

Figure S8. Quantitative RT-PCR analysis of small intestinal organoids derived from *Apc*$^{flox14/flox14}$ *Tcf4*$^{+/+}$ *Lgr5-CreERT2* (*Apc*$^{KO/KO}$ *Tcf4*$^{+/+}$) and *Apc*$^{flox14/flox14}$ *Tcf4*$^{flox5/flox5}$ *Lgr5-CreERT2* (*Apc*$^{KO/KO}$ *Tcf4*$^{KO/KO}$) mice. Total RNA was isolated from four (parallel) organoid cultures 48 hours after 4-OHT or ethanol addition to the culture media. Diagrams show relative expression levels of the indicated genes in ethanol-treated when compared to 4-OHT-treated organoids of the same genotype. RNA levels were normalized to the *Actb* mRNA levels; *Ubb* represents an additional housekeeping gene. Error bars indicate SDs.

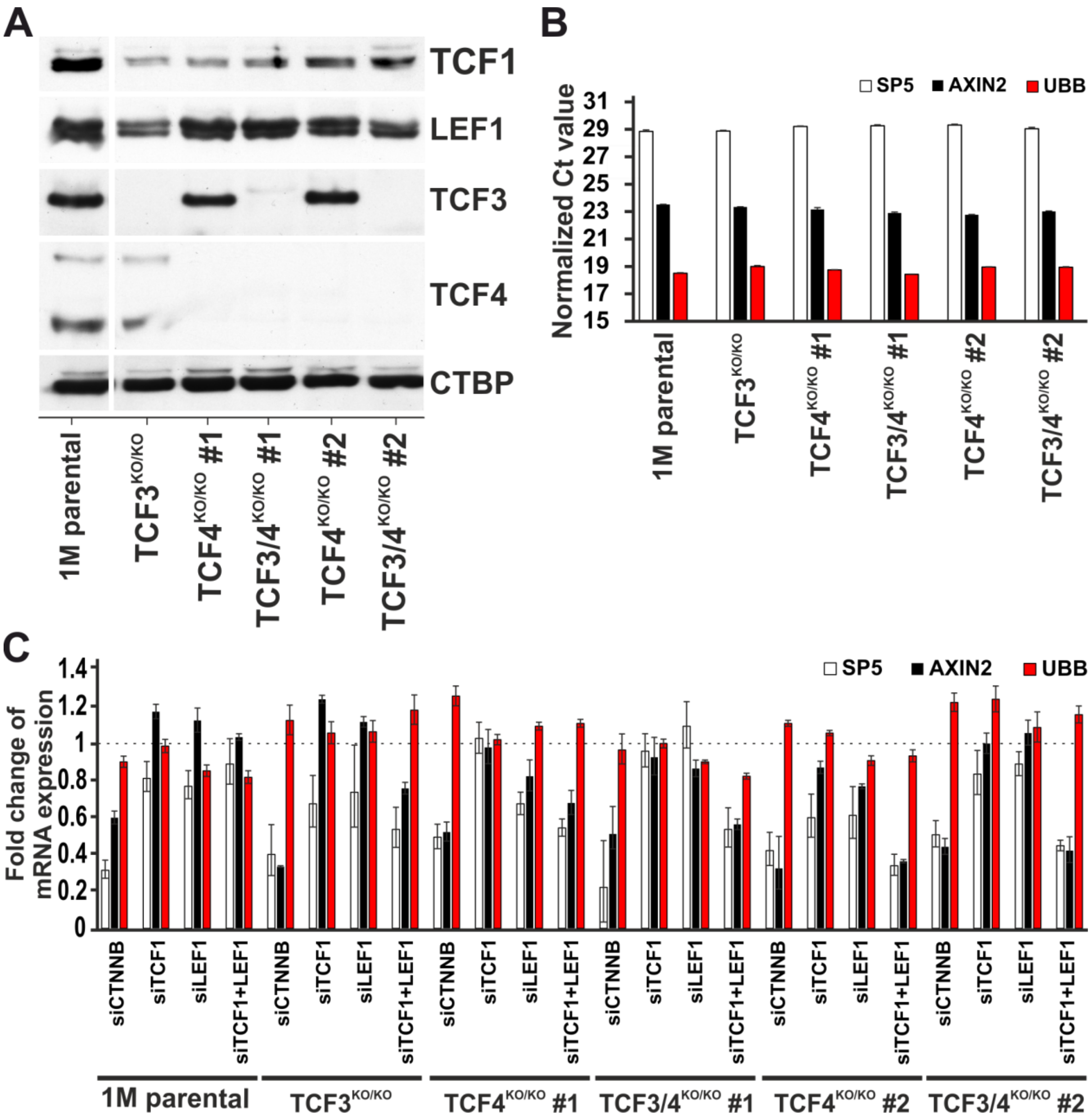

Figure S9. TCF4 is dispensable for TCF/β-catenin-dependent transcription in M1 human cells. (**A**) Western blot analysis of whole-cell lysates prepared from parental M1 cells or TCF3/TCF4 single- or double-deficient cell clones generated by CRISPR/Cas9 targeting. CTBP, control immunoblotting with C-terminal Binding Protein (CTBP)-specific antibody. (**B**) Quantitative RT-PCR analysis of Wnt signaling target genes *AXIN2* and *SP5* in indicated cell clones. The diagram shows the cycle threshold (Ct) values upon normalization to *ACTB* expression. (**C**) Quantitative RT-PCR analysis of *AXIN2* and *SP5* in indicated cells upon siRNA-mediated knockdown of TCF1 and/or LEF1 expression. CTNNB-specific siRNA was used to monitor the sensitivity of the selected genes to Wnt pathway downregulation. RNA levels were normalized to *ACTB* expression. The expression level of a given gene in the cells treated with control non-silencing siRNA (siCTRL) was set to 1. The experiments in (A) and (C) were performed twice (each experiment in triplicates); representative results are shown. *UBB* represents an additional housekeeping gene; error bars indicate SDs.

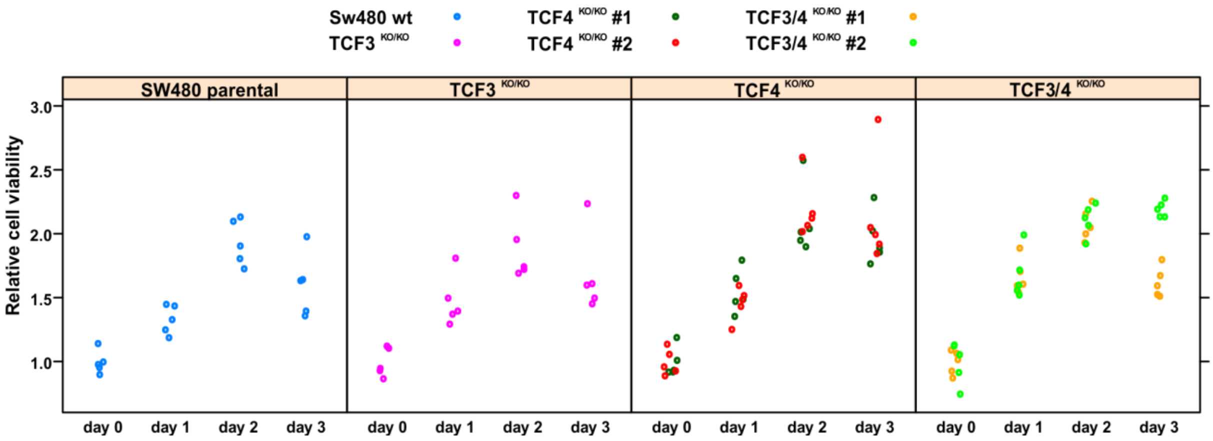

Figure S10. Cell viability test of parental SW480 cells or TCF3/TCF4 single- or double-deficient cell clones. Cells were plated in five replicates (for each individual clone) at approximately 20% confluency in a 96-well culture plate. Cell viability was measured 4 hours after the plating (day 0). The measurement was repeated every 24 hours for the following three days. Measured values were normalized to the median value obtained for day 0.

Table S1. Oligonucleotide primers used in the study.

qPCR primers		
**Gene symbol**	**Organism**	**Sequence**
Actb	Mouse	Forward: 5'-GATCTGGCACCACACCTTCT-3'
		Reverse: 5'-GGGGTGTTGAAGGTCTCAAA-3'
Axin1	Mouse	Forward: 5'-ACCCAGTACCACAGAGGACG-3'
		Reverse: 5'-CTGCTTCCTCAACCCAGAAG-3'
Axin2	Mouse	Forward: 5'-TAGGCGGAATGAAGATGGAC-3'
		Reverse: 5'-CTGGTCACCCAACAAGGAGT-3'
Alpi	Mouse	Forward: 5'-GCTGACCACTCCCATGTCTT-3'
		Reverse: 5'-TCTGAGCTTCGGTGACATTG-3'
Cccnd1	Mouse	Forward: 5'-AGTGCGTGCAGAAGGAGATT-3'
		Reverse: 5'-CTCTTCGCACTTCTGCTCCT-3'
CD24	Mouse	Forward: 5'-TTCCCCAAATCCAAGTAACG-3'
		Reverse: 5'-CACATTGGACTTGTGGTTGC-3'
Cryptdins	Mouse	Forward: 5'-AGGAGCAGCCAGGAGAAG-3'
		Reverse: 5'-ATGTTCAGCGACAGCAGAG-3'
GAPDH	Mouse	Forward: 5'-GTGGAAGCTGATCTCCAAGG-3'
		Reverse: 5'-CCTCCACGTTGACATTGTTG-3'
GFP	Mouse	Forward: 5'-GACGTAAACGGCCACAAGTT-3'
		Reverse: 5'-GAACTTCAGGGTCAGCTTGC-3'
Lef1	Mouse	Forward: 5'-TCACTGTCAGGCGACACTTC-3'
		Reverse: 5'-ATGAGGTCTTTTGGGCTCCT-3'
Lgr5	Mouse	Forward: 5'-CCTGTCCAGGCTTTCAGAAG-3'
		Reverse: 5'-CTGTGGAGTCCATCAAAGCA-3'

Mki67	Mouse	Forward: 5'-GTAACCTGCCTGCGAAGAGA-3'
		Reverse: 5'-TTTCGCAACTTTCGTTTGTG-3'
Olfm4	Mouse	Forward: 5'-TGGGCAGAAGGTGGGACTGTGT-3'
		Reverse: 5'-TGTCAGCGGGAAAGGCGGTA-3'
Tcf1 (Tcf7)	Mouse	Forward: 5'-GCCAGAAGCAAGGAGTTCAC-3'
		Reverse: 5'-TACACCAGATCCCAGCATCA-3'
Tcf3 (Tcf7l1)	Mouse	Forward: 5'-CGGGACAACTATGGGAAGAA-3'
		Reverse: 5'-TTGCTCTTAGAGGCCAGAGC-3'
Tcf4 (Tcf7l2)	Mouse	Forward: 5'-CGTAGACCCCAAAACAGGAAT-3'
		Reverse: 5'-TCCTGTCGTGATTGGGTACA-3'
Ubb	Mouse	Forward: 5'-ATGTGAAGGCCAAGATCCAG-3'
		Reverse: 5'-TAATAGCCACCCCTCAGACG-3'
ACTB	Human	Forward: 5'- GGCATCCTCACCCTGAAGTA-3'
		Reverse: 5'- AGGTGTGGTGCCAGATTTTC-3'
AXIN2	Human	Forward: 5'- CTGGCTTTGGTGAACTGTTG-3'
		Reverse: 5'- AGTTGCTCACAGCCAAGACA-3'
CTNNB1	Human	Forward: 5'- TTCCAGACACGCTATCATGC-3'
		Reverse: 5'- AATCCACTGGTGAACCAAGC-3'
LEF1	Human	Forward: 5'- CTGCTAGAGACGCTGATCCA-3'
		Reverse: 5'-TGGCTCTTGCAGTAGACGAA-3'
SP5	Human	Forward: 5'- GAAACAGTGCTCGGGTTTTC-3'
		Reverse: 5'- TAGCTCTGCATGGAGCTGAA-3'
TCF1 (TCF7)	Human	Forward: 5'- CCTCTGCCTCCCTAGCTTTT-3'
		Reverse: 5'- ATGGGGGAGATGGGTAGAGA-3'
TCF3 (TCF7L1)	Human	Forward: 5'- TCGATGTCCAGCCTGGTCTC-3'

		Reverse: 5'- GACTGGTGATTTCACGCTCA-3'
TCF4 (TCF7L2)	Human	Forward: 5'- CTCCTCATCAATTGCACAGC-3'
		Reverse: 5'- GGAGCTGTGGGAATGTAACC-3'
UBB	Human	Forward: 5'- GCTTTGTTGGGTGAGCTTGT-3'
		Reverse: 5'- TCACGAAGATCTGCATTTTGA-3'
**CRISPR guide sequences**		
**Targeted gene symbol**		**Sequence (PAM)**
TCF3 (TCF7L1)		5'- TCAGCTCGTCGTTCGCCCCG(AGG)-3'
TCF4 (TCF7L2)		5'-CCTCCACCGCCGTTCAGCTG(CGG)-3'
**N-terminal Tcf4 portion cloning PCR primers**		
**Primer**		**Sequence**
Forward primer		5'- TTTAAGCTTAAAATGCCGCAGCTGAACGGCGGT-3'
Reverse primer		5'- ACTGAATTCGAAACGATGTGTGCTGGCGACG-3'
**qPCR primers**		
**Gene symbol**	**Organism**	**Sequence**
Actb	Mouse	Forward: 5'-GATCTGGCACCACACCTTCT-3'
		Reverse: 5'-GGGGTGTTGAAGGTCTCAAA-3'
Axin1	Mouse	Forward: 5'-ACCCAGTACCACAGAGGACG-3'
		Reverse: 5'-CTGCTTCCTCAACCCAGAAG-3'
Axin2	Mouse	Forward: 5'-TAGGCGGAATGAAGATGGAC-3'
		Reverse: 5'-CTGGTCACCCAACAAGGAGT-3'
Alpi	Mouse	Forward: 5'-GCTGACCACTCCCATGTCTT-3'
		Reverse: 5'-TCTGAGCTTCGGTGACATTG-3'
Cccnd1	Mouse	Forward: 5'-AGTGCGTGCAGAAGGAGATT-3'

		Reverse: 5'-CTCTTCGCACTTCTGCTCCT-3'
CD24	Mouse	Forward: 5'-TTCCCCAAATCCAAGTAACG-3'
		Reverse: 5'-CACATTGGACTTGTGGTTGC-3'
Cryptdins	Mouse	Forward: 5'-AGGAGCAGCCAGGAGAAG-3'
		Reverse: 5'-ATGTTCAGCGACAGCAGAG-3'
GAPDH	Mouse	Forward: 5'-GTGGAAGCTGATCTCCAAGG-3'
		Reverse: 5'-CCTCCACGTTGACATTGTTG-3'
GFP	Mouse	Forward: 5'-GACGTAAACGGCCACAAGTT-3'
		Reverse: 5'-GAACTTCAGGGTCAGCTTGC-3'
Lef1	Mouse	Forward: 5'-TCACTGTCAGGCGACACTTC-3'
		Reverse: 5'-ATGAGGTCTTTTGGGCTCCT-3'
Lgr5	Mouse	Forward: 5'-CCTGTCCAGGCTTTCAGAAG-3'
		Reverse: 5'-CTGTGGAGTCCATCAAAGCA-3'
Mki67	Mouse	Forward: 5'-GTAACCTGCCTGCGAAGAGA-3'
		Reverse: 5'-TTTCGCAACTTTCGTTTGTG-3'
Olfm4	Mouse	Forward: 5'-TGGGCAGAAGGTGGGACTGTGT-3'
		Reverse: 5'-TGTCAGCGGGAAAGGCGGTA-3'
Tcf1 (Tcf7)	Mouse	Forward: 5'-GCCAGAAGCAAGGAGTTCAC-3'
		Reverse: 5'-TACACCAGATCCCAGCATCA-3'
Tcf3 (Tcf7l1)	Mouse	Forward: 5'-CGGGACAACTATGGGAAGAA-3'
		Reverse: 5'-TTGCTCTTAGAGGCCAGAGC-3'
Tcf4 (Tcf7l2)	Mouse	Forward: 5'-CGTAGACCCCAAAACAGGAAT-3'
		Reverse: 5'-TCCTGTCGTGATTGGGTACA-3'
Ubb	Mouse	Forward: 5'-ATGTGAAGGCCAAGATCCAG-3'
		Reverse: 5'-TAATAGCCACCCCTCAGACG-3'

ACTB	Human	Forward: 5'- GGCATCCTCACCCTGAAGTA-3'
		Reverse: 5'- AGGTGTGGTGCCAGATTTTC-3'
AXIN2	Human	Forward: 5'- CTGGCTTTGGTGAACTGTTG-3'
		Reverse: 5'- AGTTGCTCACAGCCAAGACA-3'
CTNNB1	Human	Forward: 5'- TTCCAGACACGCTATCATGC-3'
		Reverse: 5'- AATCCACTGGTGAACCAAGC-3'
LEF1	Human	Forward: 5'- CTGCTAGAGACGCTGATCCA-3'
		Reverse: 5'-TGGCTCTTGCAGTAGACGAA-3'
SP5	Human	Forward: 5'- GAAACAGTGCTCGGGTTTTC-3'
		Reverse: 5'- TAGCTCTGCATGGAGCTGAA-3'
TCF1 (TCF7)	Human	Forward: 5'- CCTCTGCCTCCCTAGCTTTT-3'
		Reverse: 5'- ATGGGGGAGATGGGTAGAGA-3'
TCF3 (TCF7L1)	Human	Forward: 5'- TCGATGTCCAGCCTGGTCTC-3'
		Reverse: 5'- GACTGGTGATTTCACGCTCA-3'
TCF4 (TCF7L2)	Human	Forward: 5'- CTCCTCATCAATTGCACAGC-3'
		Reverse: 5'- GGAGCTGTGGGAATGTAACC-3'
UBB	Human	Forward: 5'- GCTTTGTTGGGTGAGCTTGT-3'
		Reverse: 5'- TCACGAAGATCTGCATTTTGA-3'

**CRISPR guide sequences**	
**Targeted gene symbol**	**Sequence (PAM)**
TCF3 (TCF7L1)	5'- TCAGCTCGTCGTTCGCCCCG(AGG)-3'
TCF4 (TCF7L2)	5'-CCTCCACCGCCGTTCAGCTG(CGG)-3'

**N-terminal Tcf4 portion cloning PCR primers**	
**Primer**	**Sequence**
Forward primer	5'- TTTAAGCTTAAAATGCCGCAGCTGAACGGCGGT-3'

Reverse primer	5'- ACTGAATTCGAAACGATGTGTGCTGGCGACG-3'
